# The enigmatic 1693 AD tsunami in the eastern Mediterranean Sea: new insights on the triggering mechanisms and propagation dynamics

**DOI:** 10.1038/s41598-022-13538-x

**Published:** 2022-06-10

**Authors:** Giovanni Scicchitano, Salvatore Gambino, Giovanni Scardino, Giovanni Barreca, Felix Gross, Giuseppe Mastronuzzi, Carmelo Monaco

**Affiliations:** 1 Dipartimento di Scienze della Terra e Geoambientali, Università degli Studi di Bari Aldo Moro, 70125 Bari, Italy; 2 Interdepartmental Research Center for Coastal Dynamics, University of Bari Aldo Moro, 70125 Bari, Italy; 3 Dipartimento di Scienze Biologiche, Geologiche e Ambientali, Università degli Studi di Catania, 95129 Catania, Italy; 4 CRUST-Interuniversity Center for 3D Seismotectonics with Territorial Applications, 66100 Chieti Scalo, Italy; 5 Institute of Geosciences, Kiel University, Kiel, Germany; 6 Center for Ocean and Society, Kiel University, Kiel, Germany; 7 Istituto Nazionale di Geofisica e Vulcanologia, Osservatorio Etneo, 95131 Catania, Italy

**Keywords:** Environmental impact, Natural hazards

## Abstract

The disastrous earthquake of 1693 AD caused over 60,000 causalities and the total destruction of several villages and towns in south-eastern Sicily. Immediately after the earthquake, a tsunami struck the Ionian coasts of Sicily and the Messina Strait and was probably recorded even in the Aeolian Islands and Malta. Over the last few decades, the event has been much debated regarding the location of the seismogenic source and the possible cause of the associated tsunami. The marine event has been related to both a submarine landslide and a coseismic displacement at the seafloor. To better define the most reliable sources and dynamics of the tsunami, we couple high-resolution marine seismic survey data with hydrodynamic modelling to simulate various scenarios of tsunami generation and propagation. Results from the simulations are compared with geomorphological evidence of past tsunami impacts, described in previous work along the coast of south-eastern Sicily, and within historical chronicles and reports. The most reliable scenario considers the 1693 event composed by two different tsunami waves: a first wave generated by the coseismic fault displacement at the seafloor and a second wave generated by a submarine landslide, triggered by the earthquake shaking. Tsunami modelling shows that a simultaneous movement between fault displacement and submarine mass movement could determine a destructive interference on the tsunami waves, resulting in a reduction in wave height. For this reason, the second tsunami wave probably occurred with a maximum delay of few minutes after the one generated by the earthquake and induced a greater flooding. The double-source model could explain the observation because in the course of other destructive earthquakes in south-eastern Sicily, such as that of 1169 AD, the associated tsunami caused less damages. This implies the need to better map, define and assess the hazard responsible for this type of tsunami events.

## Introduction

**Figure 1:**
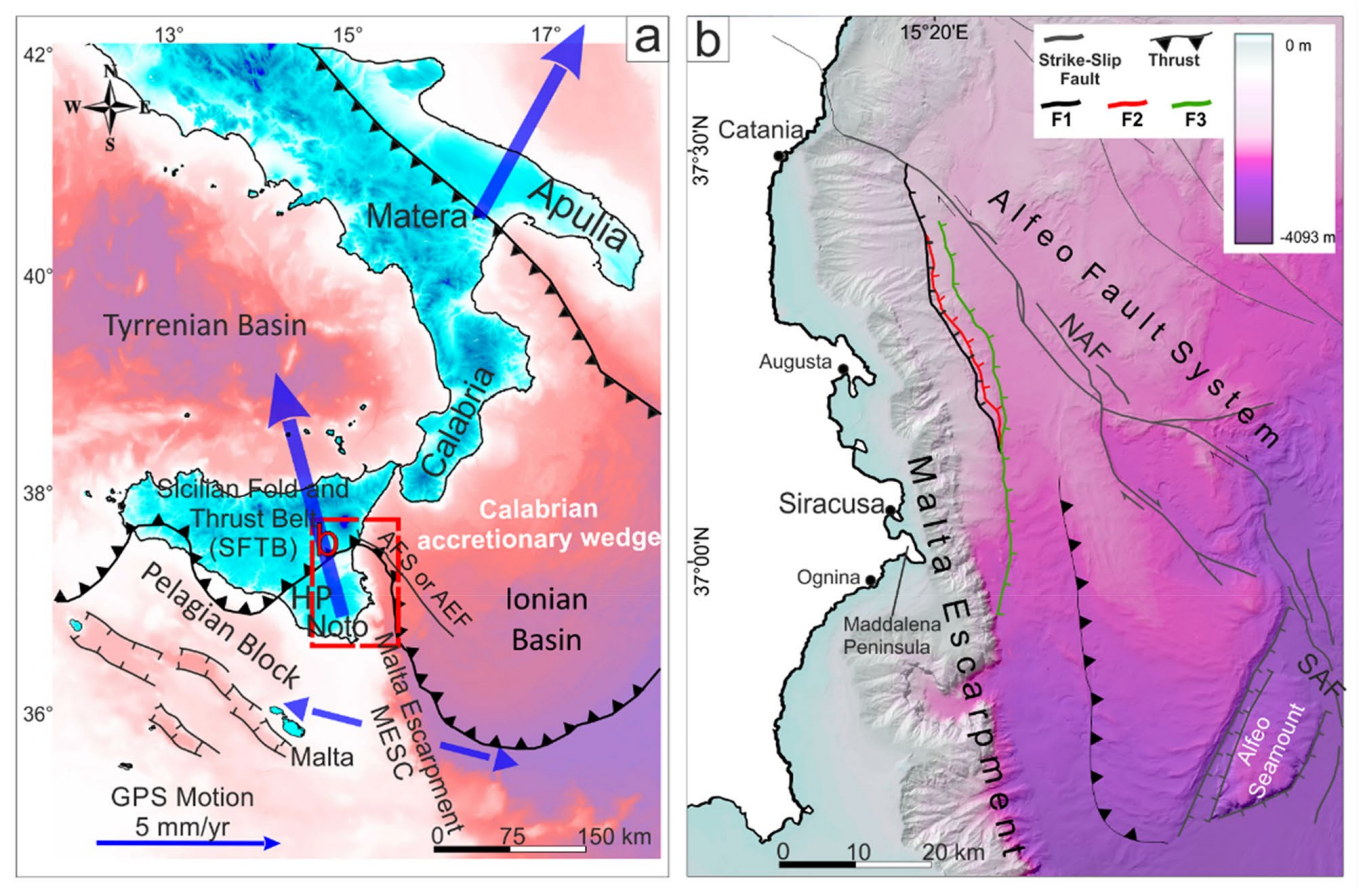
(**a**) Tectonic sketch map of southern Italy. Large blue arrows indicate the GPS vectors (see^14–16^, modified after Gambino et al.^26^) in the lower plate of the collisional system whereas small blue arrows is the resultant extension in the Western Ionian Basin. AFS, Alfeo Fault System^17,18^; AEF, Alfeo-Etna Fault^19^; (**b**) main tectonic structures of Western Ionian basin. NAF, North Alfeo Fault; SAF, South Alfeo Fault (modified after Gambino et al.^20^). The maps were obtained by co-authors through QGIS—software (version 3.14.16); https://www.qgis.org/it/site/, license Creative Commons. Attribution-Share Alike 3.0 licence (CC BY-SA) integrated with ESRI World Imagery.

**Figure 2:**
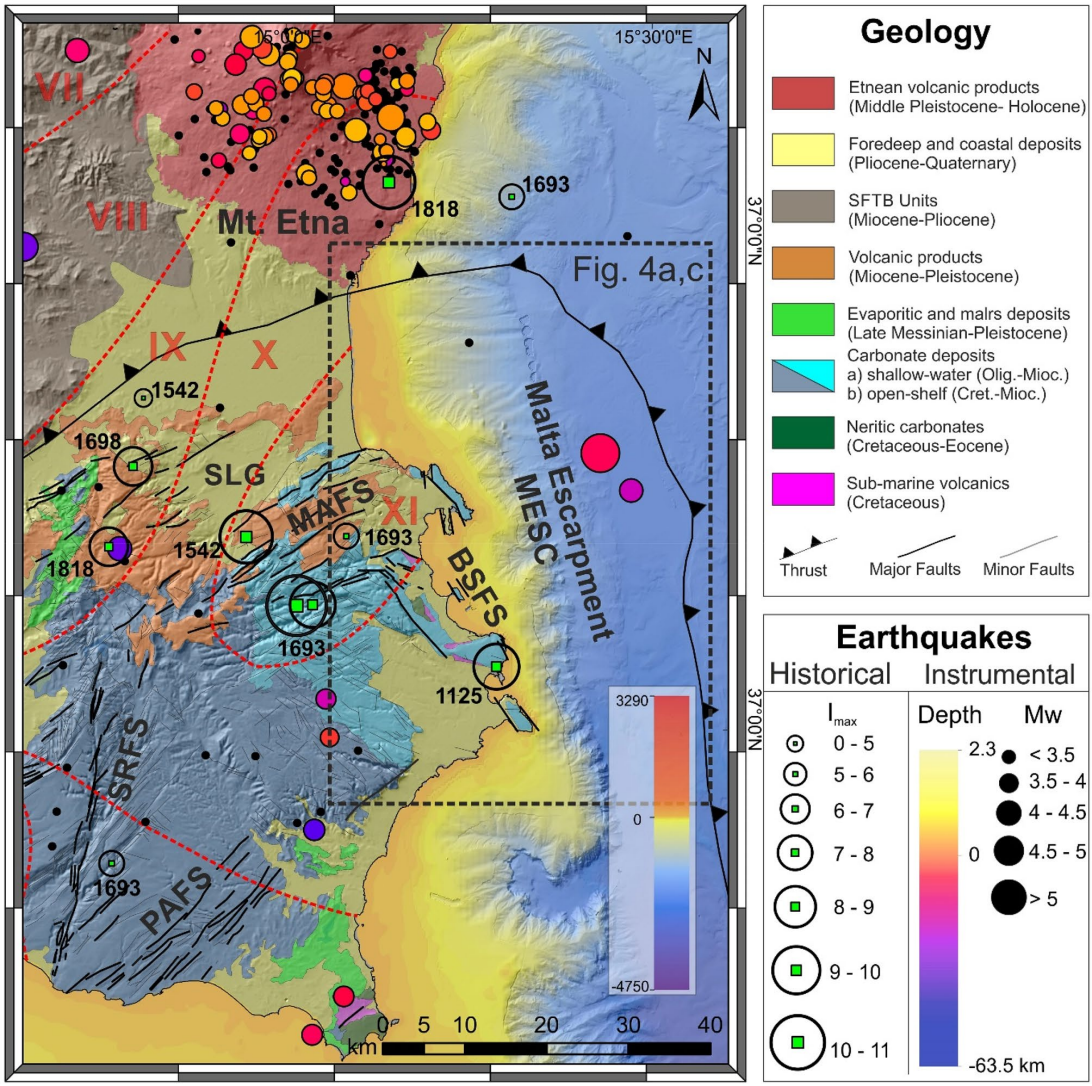
Structural setting of the Hyblean Plateau with major faults system (MAFS, Monterosso–Agnone Faults System; SRFS, Scicli–Ragusa Faults System; PAFS, Pozzallo–Avola Faults System; BSFS, Brucoli–Siracusa Faults Systems; SLG, Scordia Lentini Graben) with the location (empty circles) of Historical earthquakes (from CPTI 15^21^), and instrumental seismicity with M > 2.5 events in the period 1981–2014 (from http://istituto.ingv.it/index.php/it/archivi-e-banche-dati—see also Scarfi et al.^22^). Red dashed lines refer to the isoseismal map proposed by Barbano^23^ for the January 11, 1693 earthquake. The map was obtained by co-authors through QGIS—software (version 3.14.16); https://www.qgis.org/it/site/, license Creative Commons. Attribution-Share Alike 3.0 licence (CC BY-SA) integrated with ESRI World Imagery.

The Mediterranean Sea experienced several tsunami events, as testified by historical records and geological evidence. The geodynamic activity of the Mediterranean basin has determined the occurrences of tsunami that have been related to seismic and non-seismic sources^1–3^. The non-seismic or mixed seismic/non-seismic sources have also recently generated significant tsunamis in the Mediterranean, such as that generated by a landslide at Stromboli in 2002^4–8^, and that related to the Mw 7.1 earthquake and submarine landslide of the Strait of Messina in 1908^9–12^. The Western Ionian basin (eastern Sicily and southern Calabria in particular—Fig. 1) is one of the most seismically active sectors of the Italian peninsula and within the central Mediterranean. Several seismic events with estimated moment magnitude greater than 7 have struck eastern Sicily. Most of these have been associated with tsunami generation (e.g. the 1169, 1542, 1693, 1818, 1908, 1990 events—ITC, Italian Tsunami Catalogue^13^). Excluding the 1908 event, the seismogenic sources of these large earthquakes have been located in the Hyblean Plateau or in neighboring areas (south-eastern Sicily, Fig. 2).

The strongest tsunami described in the Mediterranean was associated with the January 11, 1693 seismic event (M ≈ 7.3), whose source is still a matter of debate. Some authors proposed an offshore fault displacement on the seafloor as the most plausible source of the tsunami and identified, through marine geophysical campaigns, distinct possible causative tectonic structures^24–26^. Recently, tsunami propagation and impact on the coast of Ognina, an area located 30 km south of the town of Siracusa (Fig. 1), was modelled by Scardino et al.^27^, considering distinct seismic events, including that of 1693, and distinct fault scarps on the seafloor as the source of the tsunami waves. According to these authors, the fault proposed by Gambino et al.^26^ is the most plausible for the generation of tsunamis with associated waves able to reach the southern coastal sector of south-eastern Sicily. Other authors instead considered a marine landslide triggered by the earthquake as a possible source of the 1693 tsunami^28–30^.

However, dynamics related to the propagation of two distinct tsunamis, one generated by a fault displacement and the other one generated by a submarine landslide, have never been considered and modelled.

## Geological settings

South-eastern Sicily (the Hyblean Plateau in Fig. 1) represents the emergent portion of the Pelagian Block, a foreland domain along the northern margin of the African plate^31^. The 25–30 km thick Hyblean crust is covered by a 5–8 km thick carbonate succession, consisting of a sequence of shallow to deep-water Meso-Cenozoic rocks, with intercalations of sub-marine to sub-aerial volcanic products dated between the Cretaceous and the Pleistocene. Quaternary sedimentary deposits are generally preserved within structural depressions at the edge of the Hyblean Plateau^32,33^.

The geological record indicates a Plio-Pleistocene extensional tectonic deformation of the Hyblean Plateau, resulting in NW–SE and SW-NE normal fault systems^32–34^. As revealed by space borne geodetic data^15,35^, boreholes stress measurements^36^, and seismogenic stress tensors^37^, the Hyblean region is currently undergoing a sub-horizontal NNW oriented max stress causing local reactivation of previous NW–SE trending extensional faults as strike-slip structures or positive tectonic inversion along ENE-WSW oriented structures (see Cultrera et al.^32^), consistent with the Africa‐Eurasia plate convergence dynamics. The eastern border of the Hyblean Plateau is characterized by the Malta ESCarpment (hereinafter MESC), a 300-km long crustal and bathymetric discontinuity that marks the transition between the Hyblean continental crust and the adjacent Ionian oceanic Basin^38^. The MESC formed since the Permo-Triassic in response to crustal stretching^39^ and to the subsequent Jurassic–Cretaceous spreading stage^17,38^. Successively, during the Plio–Quaternary, the discontinuity was reactivated by normal-oblique extension^26,16^ in the context of the convergence between Africa and Eurasia plates. The recent activity of the MESC has been characterized by slow vertical deformation rates that, combined with sea-level changes, generated several orders of marine terraces and paleo-shorelines in the uplifted footwall block along the adjacent coastal area^40–44^.

The late Quaternary sense of motion along the MESC is still debated (Fig. 1a). Past geological-structural observations^45^ and seismological data^37^ seem to suggest that the onshore structures related to the MESC fault system are characterized by left-lateral kinematics. Conversely, recent field studies^17^, seismological^46^ and geodetic data^16^, suggest a right-lateral component of motion along the MESC fault system. This kinematics is consistent with diverging GPS vectors measured on the lower plate of the collisional system (i.e., the Hyblean and Adria blocks^14,15^), that indicates a crustal extension along the ESE–WNW direction. In this geodynamic context, the NNW–SSE oriented MESC should be reactivated obliquely according to right-lateral transtensional kinematics. Marine seismic profiles confirm active extension along the northern sector of the MESC^24,26,28,17^. In this frame, the reactivation of the MESC has been also interpreted as related to the vertical detachment of the Ionian slab or as part of a regional scale lithospheric belt accommodating differential motion of adjacent Western Ionian compartments, and/or connecting the thrust zone along the northern margin of Sicily with the Calabrian subduction^16,19^. East of the MESC, the down-faulted Western Ionian Basin and the tectonically overlain Calabrian accretionary prism are deformed by a large-scale right-lateral shear zone (Alfeo Fault System, Fig. 1^17,19^), interpreted as the surface expression of the retreating Ionian slab^47^. Whatever the geodynamic process producing active deformation along the Western Ionian Basin, the resulting structural features must be considered in the assessment of seismic hazard of the region. Both the MESC and the North Alfeo fault (Fig. 1b), due to their dimension and active deformation, have been considered reliable sources of destructive earthquakes^24,25,17,47^. However, considering the almost pure strike-slip kinematics characterizing the NAF^17^, tsunami generation is unlikely along it. Conversely, a coseismic slip on the reactivated sector of the MESC^26^ is here considered more reliable.

## Historical earthquakes and tsunami constraints in South-Eastern Sicily

Large earthquakes and tsunamis struck eastern Sicily both in instrumental and pre-instrumental times (Fig. 2b; Table 1). Minor events, with a relatively limited magnitude, need to be mentioned for their impact, such as the December 10, 1542 (M ≈ 6.6), the October 03, 1624 (M ≈ 5.5), the February 20, 1818 (M ≈ 6.3), the January 11, 1848 (M ≈ 5.5), and lastly, the instrumentally recorded December 13, 1990 earthquake (M = 5.6).

**Table 1:** Earthquake-related tsunami impacting the coasts of south-eastern Sicily in historical times, with relative moment magnitude (Mw).

Event	Date	Geographical area	Earthquake features (estimated)	References
Earthquake and tsunami	February 4, 1169	South-eastern Sicily	Mw ≈ 6.8–7.3	Barbano et al.^48^ Tinti et al.^13^
Earthquake and tsunami	January 11, 1693	South-eastern Sicily	Mw ≈ 7.4	Barbano et al.^48^ Tinti et al.^13^ (CPTI15-vers.4, see Rovida et al.^49^)
Earthquake and tsunami	December 28, 1908	Messina Strait	Mw = 7.1	Barreca et al.^10^; Meschis et al.^11^; Schambach et al.^12^ Tinti et al.^13^

The exact location of the seismic sources for historical events is still a matter of debate and substantially unknown, in particularly with regard to the seismic sequence of 1693. Based on macroseismic intensity and morphotectonic data, it has been located on land (i) within the ENE-WSW Scordia-Lentini Graben^50^, (ii) along the Scicli-Ragusa Fault System (SRFS, Fig. 2) or (iii) along a NNW-SSE trending alignment along the eastern sector of the Hyblean Plateau (BSFS in Fig. 2). Other authors proposed a possible source in the Catania-Siracusa offshore (along the MESC, see Fig. 2), in consideration of the associated tsunami^51^. This evidence, furtherly supported by tectonics investigations through seismic profiles, led other authors to suggest, as possible seismogenic sources, offshore structures such as the MESC^24–26^ or a locked subduction fault plane^52^. It is worth noting that the 60-km long, NW–SE trending North-Alfeo Fault (NAF in Fig. 1^17^) is capable of generating Mw ≈ 7 earthquakes even if its strike-slip kinematics is not in favor of the tsunami generation.

**Figure 3:**
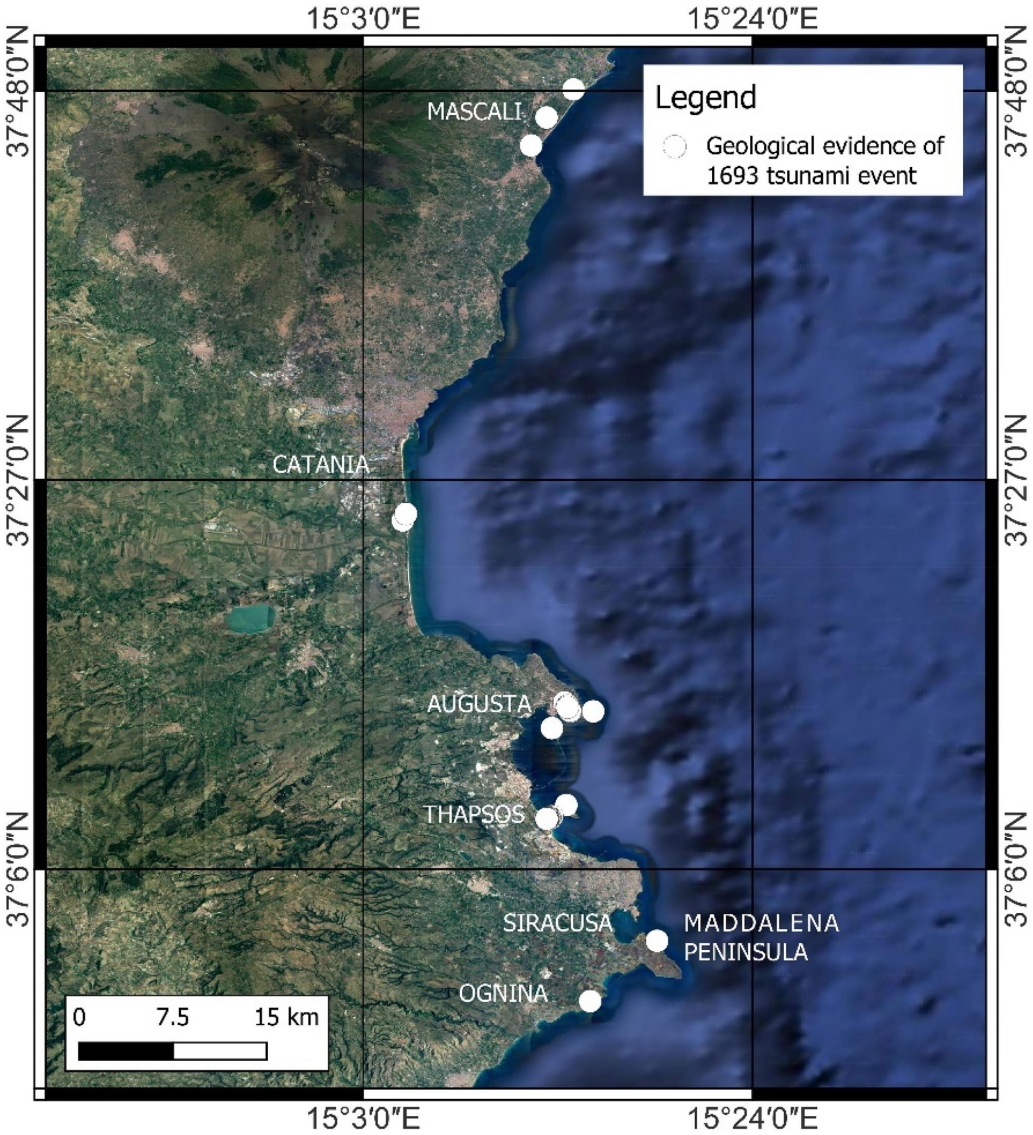
Locations of geological evidence of the 1693 tsunami event along the coast of south-eastern Sicily. The map was obtained by co-authors through QGIS—software (version 3.14.16); https://www.qgis.org/it/site/, license Creative Commons. Attribution-Share Alike 3.0 licence (CC BY-SA) integrated with ESRI World Imagery.

The aforementioned tsunamigenic events left geological evidence along the coasts of south-eastern Sicily (e.g., the 1693 tsunami, Fig. 3). Scicchitano et al.^53^ described several boulder fields located along various promontories between the towns of Augusta and Siracusa. Hydrodynamic analyses and radiocarbon dating suggested that three tsunami events were responsible for the displacements of larger boulders. Other studies^54,55^ found anomalous deposits inside coastal lagoons located between Mascali (north of Catania) and Marzamemi (south of Siracusa), attributing them to the tsunami events of 365 AD, 1693 AD and 1908 AD. Finally, Scicchitano et al.^56,57^ described in the Ognina area, 30 km south of Siracusa, a high-energy deposit, composed of a mix of marine and brackish levels, whose coarse intercalations have been interpreted as linked to distinct tsunami events. One of these events was attributed to the tsunami of 1693 AD^27,56^. Further, the historical sources reported a double withdrawal of the 1693 tsunami waves in several sectors of south-eastern Sicily, in particular in the areas of Mascali, Catania, Augusta and Priolo-Thapsos^25,58,59^.

## Tsunami modelling

**Figure 4:**
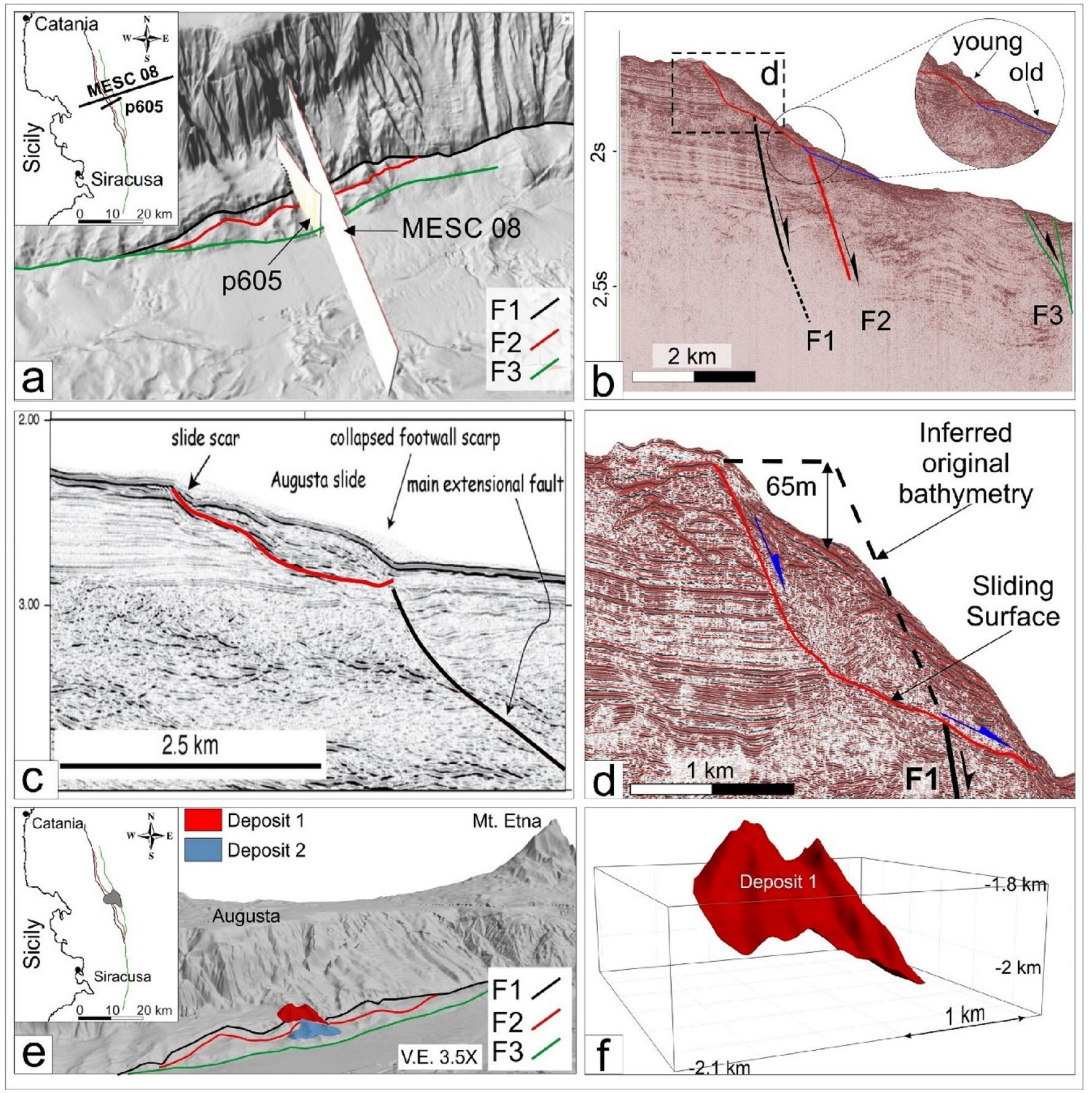
3D modelling of the submarine landslide deposits. (**a**) Location of main faults and seismic profiles (see Gambino et al.^26^) passing across the submarine landslide deposits. (**b**) P605 seismic profile showing the two identified landslide deposits offshore Augusta (red and blue lines indicate deposit 1 and 2 sliding surfaces, respectively). (**c**,**d**) Seismic expression of the most recent landslide deposit 1 along the MESC 08 (Argnani et al.^28^) and P607 profiles, respectively. (**e**) Perspective view (from SE) showing the seafloor expression of the mapped landslide deposits deriving from 3D modelling. (**f**) modelled 3D volume of deposit 1 within the MOVE software—version 2020-12.5, https://www.petex.com/products/move-suite/.

In this work, three different scenarios for the 1693 tsunami have been modelled: (i) a singular tsunami generated by fault displacement of the seafloor (hereinafter SFS—Single wave Fault Scenario), (ii) a singular tsunami generated by a submarine landslide (hereinafter SLS—Single wave Landslide Scenario), (iii) two distinct tsunamis generated by the fault displacement (the first one) and by a triggered landslide (hereinafter DTS—Double waves Tsunami Scenario). Following Scardino et al.^27^, we used for the SFS modelling the F3 fault proposed by Gambino et al.^26^, which extends offshore from Catania to the Maddalena Peninsula (Fig. 1b). During the marine seismic campaign carried out in 2016 along the Western Ionian Sea, the submarine landslide considered for the SLS modelling was detected^28,30^. This landslide is characterized by a length of 4300 m, a width of 2400 m, and a thickness of about 65 m (Table 2). It is located 15 km offshore the town of Augusta (Fig. 1b), and it is characterized by significant dimension able to generate a tsunami wave. The seismic profiles show that the submarine landslide is characterized by a variable degree of internal deformation. Reflectors appear more chaotic approaching the sliding surface where a blanket zone, possibly related to fluid circulation, is also observed (Fig. 4d). The upper part of the slipped mass is instead characterized by slightly undulated reflectors. This aspect, along with the limited movement along the MESC slope (see Fig. 4d), the preserved seafloor morphology (canyons and crests have been only translated downward), and the interpreted lithology (mostly sandstones, see Gambino et al.^20^), suggest a rigid translational movement of the mass deposit. Considering the low deformation affecting the landslide and the limited affected area, the rigid model of GEOWAVE^60^ was preferred to the rheological model^61,62^. Rigid models are usually applied to simulate the high tsunami waves caused by landslide movements of non-deformable bodies or to granular flow^4,63^.

**Table 2:** Modelled scenario in GEOWAVE with reported typology of the event, sources and their parameters.

Code	Event	Sources	Source parameters	Time step for simulation (s)
SFS	Singular tsunami	Fault F3 displacement on the sea-floor	Mean Strike: N352E Mean Dip: 49° Length: 56.46 km Width: 5.275 m Mw: 7.4 seafloor rupture:2.3 m	0.7
SLS	Singular tsunami	Submarine landslide, correlated to deposit 1, triggered by the earthquake generated by fault F3	Length: 4300 m Thickness: 65 m Density: 2700 kg/m^3^ Width: 2300 m	0.6
DTS	Double tsunami	Dual mechanism due to the fault F3 displacement and submarine landslide, correlated to deposit 1, triggered by the earthquake	Fault Mean Strike: N352E Mean Dip: 49° Length: 56.46 km Width: 5.275 m Mw: 7.4 seafloor rupture:2.3 m Landslide Length: 4300 m Thickness: 65 m Density: 2700 kg/m^3^ Width: 2300 m	0.8

The DTS modelling was obtained considering the most reliable delay between the wave front due to the fault displacement and the wave front due to landslide motion^60,64^, as deduced from historical reports^25,58^. A delay between seismic displacement and submarine landslide motion was also observed for other tsunami events around the world, such as those in Papa Nuova Guinea^65^, Palu Bay, Indonesia^66^, Grand Banks, Newfoundland^67^, Aleutian earthquake, Unimak^68^.

The tsunami flooding of the 1693 event was modelled through the GEOWAVE software^60^ in Celeris environment^69^. The GEOWAVE was used to model both the wave generation, considering the fault displacement and the submarine landslides as tsunamogenic sources, and the high-resolution bathymetry for the wave propagation from offshore to the coastline. The coastal flooding due to the tsunami impact was modelled in Celeris environment^70^, considering the shallow water bathymetry and the topography reconstructed by historical documents reporting the 1693 event. The bottom friction was considered in Celeris environment following the approach of Gibbons et al.^71,72^ and using a constant Manning value equal to 0.03, which resulted the more representative for the operational tsunami modelling on the low-lying coastal areas of south-eastern Sicily^71,73^.

## Results—tsunamogenic sources and hydrodynamic models of January 11, 1693 event

The potential sources of earthquakes and tsunamis along the Ionian coast of south-eastern Sicily have been recently described by Gambino et al.^26^. The authors reconstructed three main splays of a deeper normal fault (F1-F2-F3, Figs. 1b and 4a) and, based on the slip tendency analysis and the geometric scaling relations, concluded that all faults are prone to be reactivated according to the reconstructed stress field, even if only the F3 is potentially capable of producing an earthquake with M > 7. Accordingly, by modelling triggering and propagation of fault-generated tsunami, Scardino et al.^27^ proposed that only the F3 could have generated tsunami events comparable with those occurred in south-eastern Sicily. The submarine landslide (Fig. 4) was previously identified by Argnani et al.^28^, who estimated its areal surface of about 40 km^2^, and a volume of 4.8 km^3^. However, the lack of high-resolution bathymetry and the exploiting of a single seismic profile (MESC 08) are not sufficient to validate these estimations.

The P607 high-resolution seismic profile (Fig. 4b), which is parallel to the MESC 08 line (Fig. 4a), and the bathymetric data available (see “Materials and methods”) made it possible to better constrain the dimension and geometric characteristic of the landslide deposit previously identified (e.g. the “*Augusta slide*” of Argnani et al.^28^) through the construction of a consistent 3D model. This also allowed us to detect another older landslide deposit downslope (see inset in Fig. 4b and deposit 2 in Fig. 4e). The younger deposit (deposit 1 in Fig. 4e) is bounded at the bottom by a basal sliding surface that truncates a seismic unit characterized by high-amplitude and continuous reflectors (see Fig. 4c). The deposit seals the F1 fault (black line in Fig. 4b,c), which is considered active and characterized by high slip rate^24–26,28^ with a clear seafloor expression north and south of deposit 1 (see Fig. 4d). Considering the sedimentation rate estimated for this region (0.64–0.69 mm/yr^74^), the lack of recent sediments above deposit 1 rules out an ancient age for this landslide. For these reasons, deposit 1 is regarded as the product of a recent gravitative collapse and has been selected for the tsunami modelling. The paleo-bathymetric profile suggests a vertical drop of 65 m (Fig. 4d). 3D modelling revealed that deposit 1 has a volume of 0.45 km^3^, and an extension of 7.65 km^2^ while the volume of deposit 2 is estimated in 0.3 km^3^, and its extension in about 6.3 km^2^.

**Figure 5:**
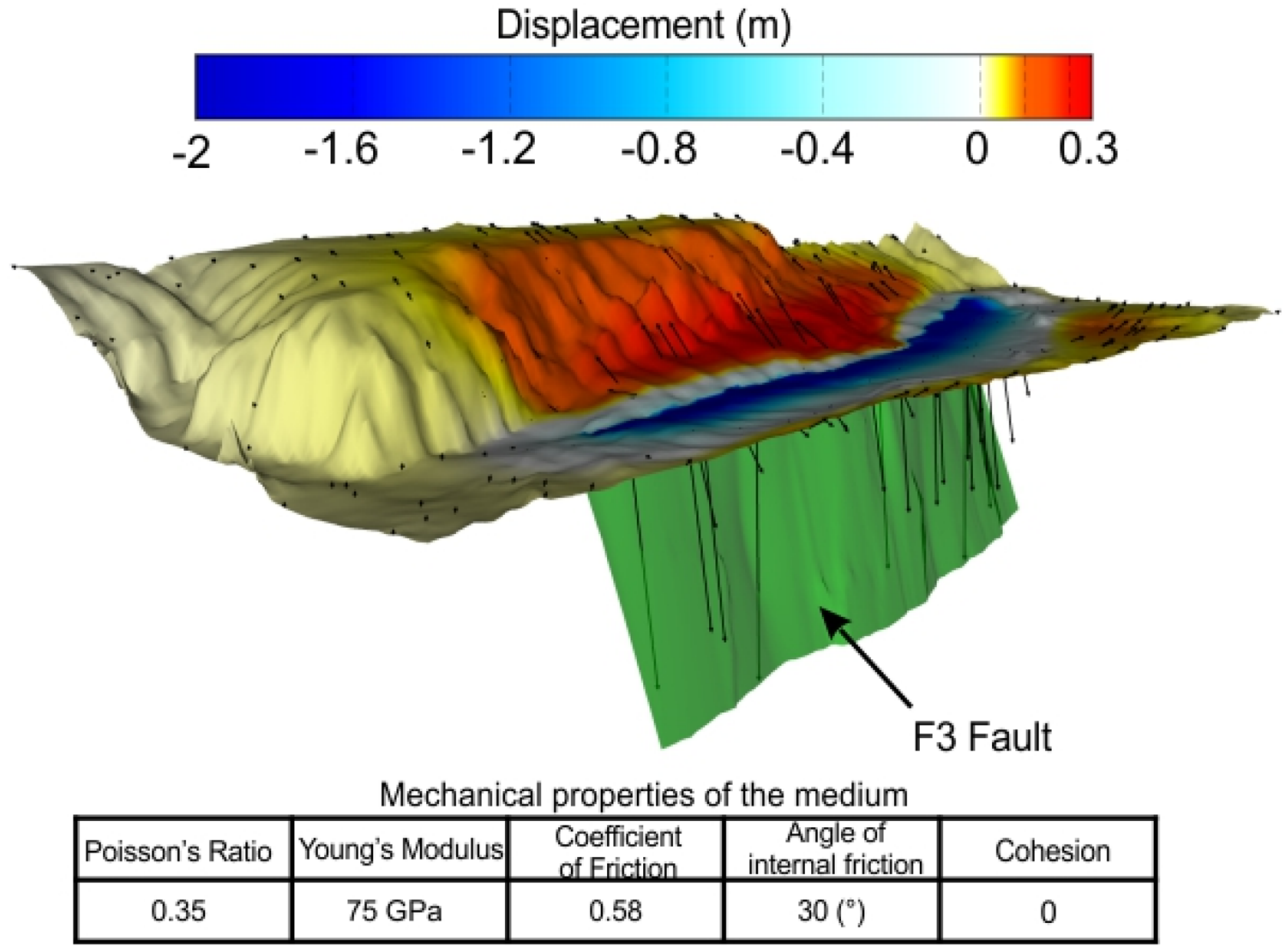
Fault response modelling and resulting displacement field achieved by simulating a distributed dip-slip motion of 4.8 m along the F3 fault-plane. The mechanical parameters of the medium surrounding the fault are listed in the table below. Black arrows are the calculated slip vectors. The figure was edited by co-authors through MOVE software—version 2020 12.5, https://www.petex.com/products/move-suite/.

The described landslides fall in the area where the maximum macroseismic Intensity (XI) for the historical earthquakes was reconstructed by Barbano et al.^48^. According to the relationships between macroseismic Intensity and Peak Groud Acceleration (PGA) (see Wald et al.^75^ among many others), an Intensity XI correspond to a PGA > 1240 Gal or to a PGV (Peak Groud Velocity) > 116 cm/s, consistent with triggering of submarine landslides. In particular, according to the CPTI15-vers.4^49^, the January 11, 1693 seismic event was a quite powerful earthquake with estimated Magnitude of 7.4 (see Table 1). Using this magnitude value and empirical scale-relationships^76^, a distributed coseismic slip of at least 4.8 m over the whole F3 fault plane is required to trigger such an intense seismic event. The magnitude of the expected slip was then exploited to perform elastic dislocation models (see Okada^77^), aimed at estimating the expected coseismic displacement of the seafloor predicted on the basis of the mechanical properties assumed for the medium involved (Table 2). Considering the stress field acting in the area (see Gambino et al.^26^), a dip-slip motion of 4.8 m was simulated along the entire F3 fault plane. A 2 m hangingwall subsidence, and a 0.3 m footwall uplift have been predicted, resulting in a 2.3 m coseismic fault-scarp at the seafloor (Fig. 5).

Three different tsunami scenarios have been modelled using the source parameters reported in Table 2.

**Figure 6:**
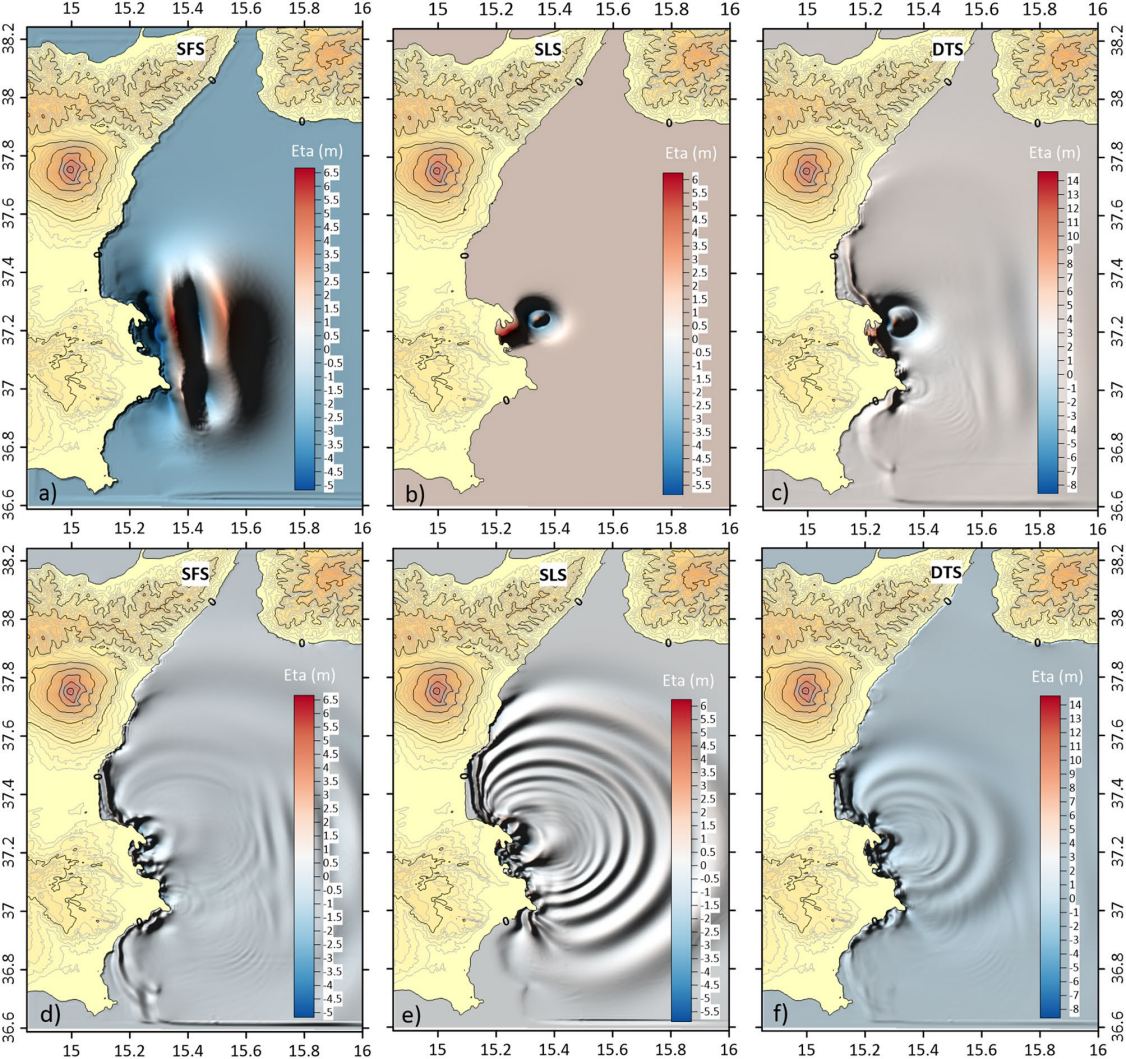
Initial sea-surface elevation (m) and wave impacting the coast under different scenarios. Simulations after 60 s of the starting event: (**a**) SFS—earthquake-generated tsunami; (**b**) SLS—submarine landslide-generated tsunami; (**c**) DTS—dual mechanism due to earthquake and submarine landslide. Simulations after 9 min of the starting event: (**d**) SFS—earthquake-generated tsunami; (**e**) SLS—submarine landslide-generated tsunami; (**f**) DTS—dual mechanism due to earthquake and submarine landslide. The maps were obtained by co-authors through QGIS—software (version 3.14.16); https://www.qgis.org/it/site/, license Creative Commons. Attribution-Share Alike 3.0 licence (CC BY-SA) integrated with ESRI World Imagery.

Multiple simulations were performed to simulate the most probable scenario according to different faulting/landslide delays. According to the three scenarios, tsunami modelling provides different dynamics of triggering, propagation and impact along the coastal areas. The triggering of the singular tsunami related to the seafloor fault displacement (SFS scenario) results in a wave that propagate with a unique front toward the coast of south-eastern Sicily, reaching heights of about 10 m (Fig. 6a), while the tsunami wave triggered by the submarine landslide (SLS scenario), shows a radial spreading^78^ that mostly impacts on the coast of Catania, Augusta, Priolo-Thapsos and Maddalena Peninsula reaching heights of about 2 m (Fig. 6b,e). The greatest value of the highest wave height was obtained for DTS scenario, with a first impacting wave 10 m high and a second 13 m high (Fig. 6c,f).

Wave height, period and direction, for the considered scenarios, were nested in Celeris environment to assess the flooding of coastal areas of eastern Sicily that were widely submerged by the 1693 tsunami wave. Six sites, for which geological and historical constrains are available (Mascali, Catania, Augusta, Priolo-Thapsos, Siracusa and Ognina, Table 3), have been selected. The run-up for each area was obtained by extracting the maximum elevations reached by floods from the modelled tsunami and from the reconstructed DTM of past topography.

**Table 3:** Inland flooding assessed through historical record, geological evidence and numerical models for the areas of Mascali, Catania, Augusta, Priolo-Thapsos, Siracusa, Ognina (see Fig. 3 for location).

Area	Historical record		Geological evidence		Flooding modelled by different scenarios (m)					
	Flooding extension fromhistorical sources (m)	Run up (m)	Flooding extension from geological evidence (m)	Run up (m)	Flooding in SFS scenario (m)	Run up (m)	Flooding in SLS scenario (m)	Run up (m)	Flooding in DTS scenario (m)	Run up (m)
Mascali	1500	–	–	10.2	150	3	100	3	1075	1
Catania	350	12	–	7.5	410	3	100	2	510	15
Augusta	165	8	790	7	670	3	500	2	960	7.5
Priolo-Thapsos	–	1.5	520	0.5	630	1	640	1	750	2
Siracusa	900	4.5			1270	1.5	950	1.5	1300	2
Ognina		–	700	5	780	5	150	2	800	5

**Figure 7:**
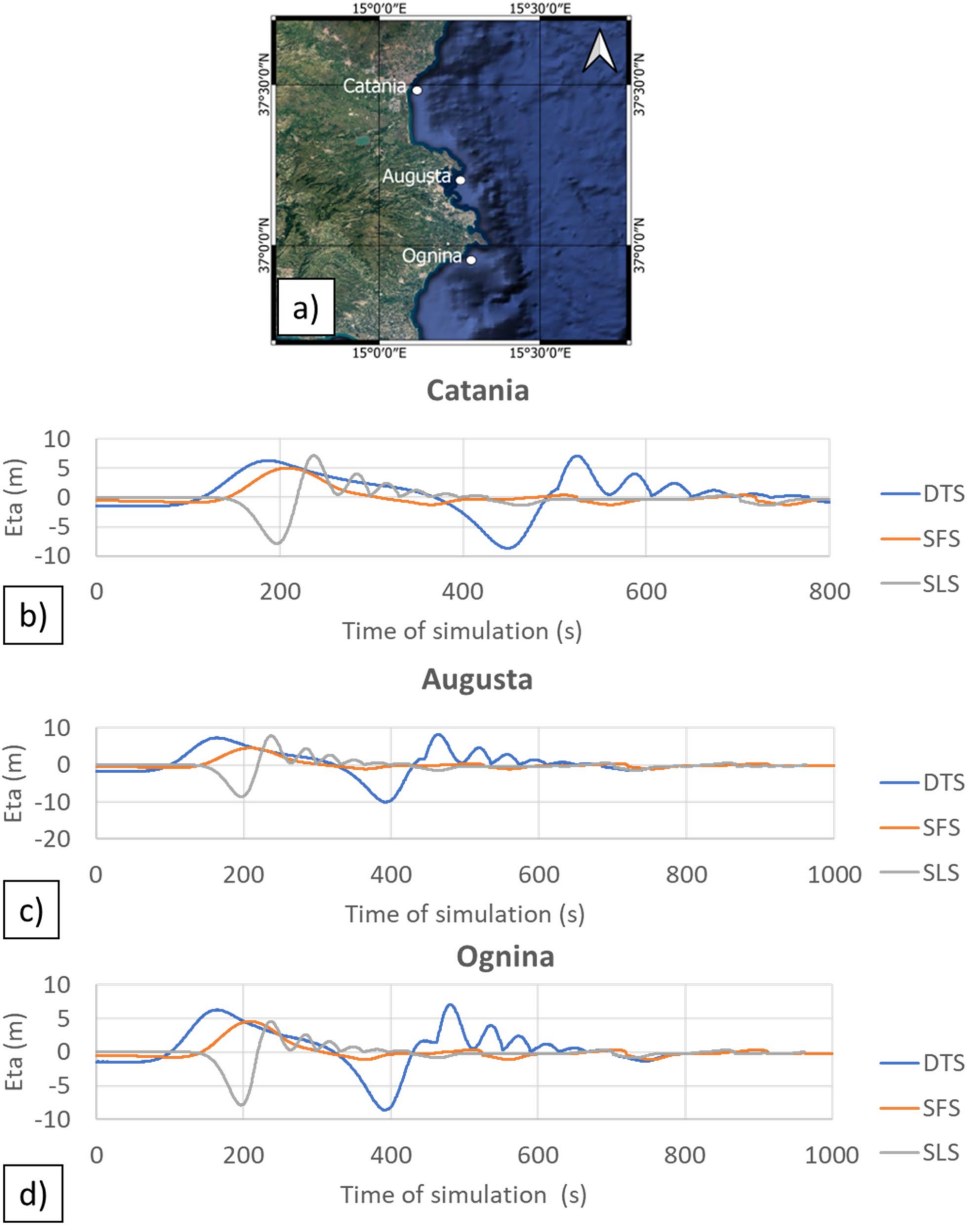
Sea-surface elevations (Eta) for three coastal areas (**a**): Catania, Augusta, and Ognina; the map was obtained by co-authors through QGIS—software (version 3.14.16); https://www.qgis.org/it/site/, license Creative Commons. Attribution-Share Alike 3.0 licence (CC BY-SA) integrated with ESRI World Imagery; (**b**) time-series for Catania; (**c**) time-series for Augusta; (**d**) time-series for Ognina. Each surface elevation was modelled for the three scenarios: DTS, SFS, SLS. The DTS scenario showed the negative trough after the first tsunami wave followed by the second tsunami wave.

**Figure 8:**
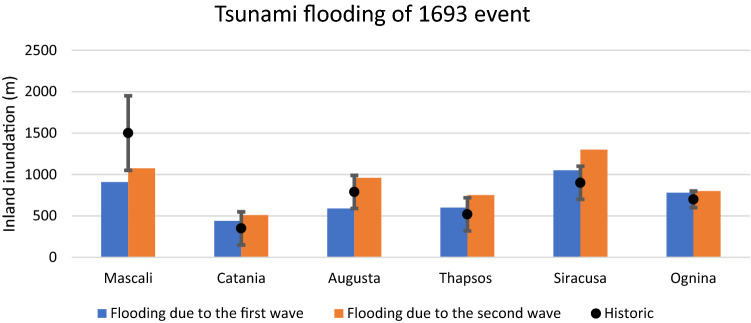
Inland inundation modelled for the coastal areas of Mascali, Catania, Augusta, Priolo-Thapsos Peninsula, Siracusa, Ognina (see Fig. 3 for location): flooding due to the first wave impact is marked with blue histogram; flooding due to the second wave impact is marked with red histogram.

**Figure 9:**
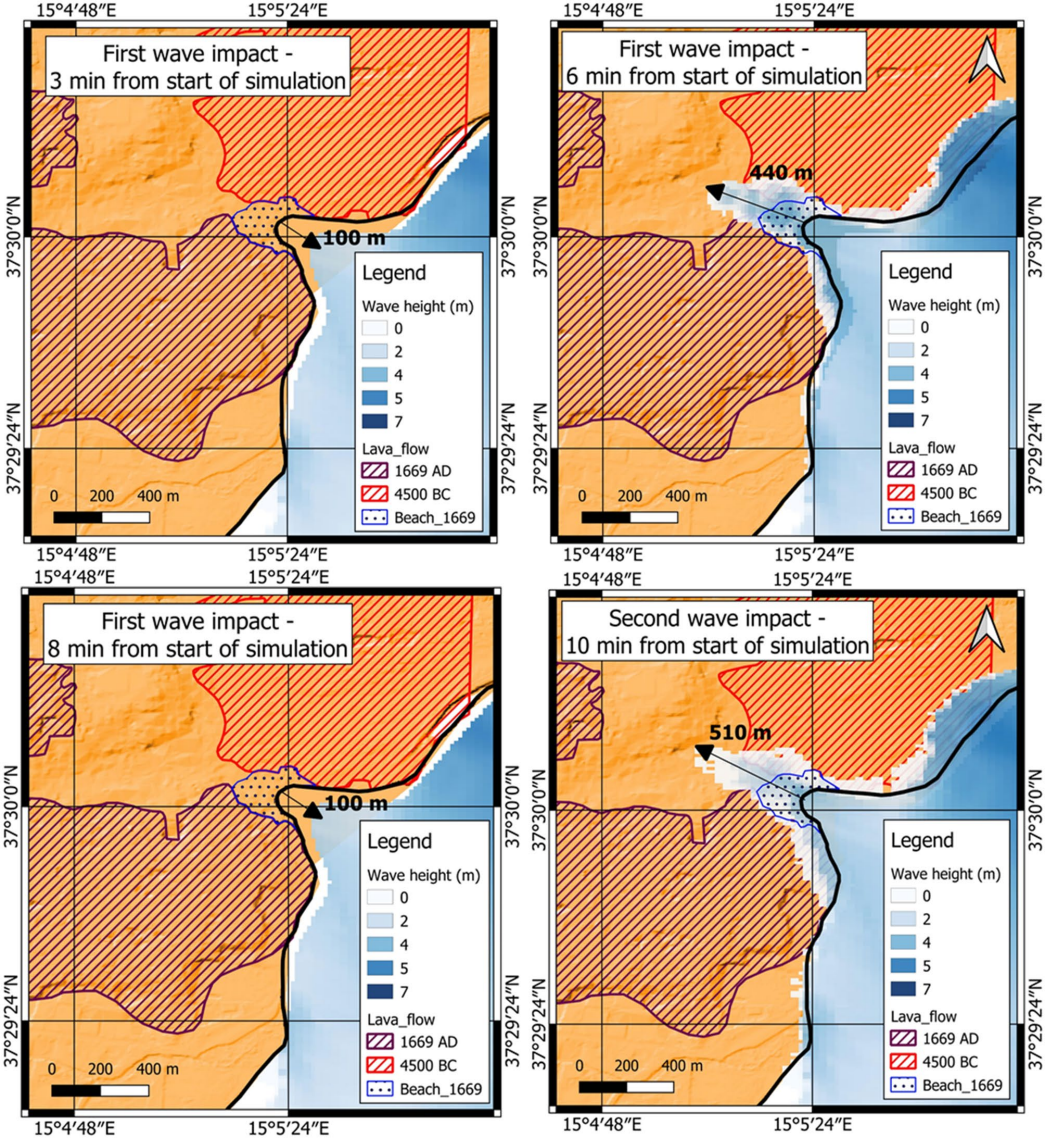
Time steps of the coastal flooding modelled in Celeris environment for the coastal area of Catania in DTS scenario. The maps were obtained by co-authors through QGIS—software (version 3.14.16); https://www.qgis.org/it/site/, license Creative Commons. Attribution-Share Alike 3.0 licence (CC BY-SA) integrated with ESRI World Imagery.

The model outputs of the double wave impact in the dual mechanism scenario (DTS) highlighted a larger inundation area than in the other two scenarios (Table 3). The initial flooding surface, generated by the tsunami triggered by the seafloor displacement predicted for the F3 fault, provided support for a second larger inundation, generated by the landslide-triggered tsunami, as showed by sea-surface elevation in Catania, Augusta, and Ognina (Fig. 7). This is due to the decrease of friction forces generated by the intense propagation of the first wave along the coastal areas. The inundations were compared with historical records and geological evidence, assuming the uncertainty of the inundation of about 50 m, as reported in the historical documents^79^, showing greater uncertainty in the low-lying coasts. This was particularly evident in the Mascali, Augusta and Siracusa areas (Fig. 8). Only for the DTS scenario the simulations showed the effect of double withdrawal when waves approached to the coasts, with linear extension of about 100 m in the Catania coastal area (Fig. 9). Similar withdrawal values were obtained also for the other coastal areas.

In 1693 AD, the Catania coast was mostly characterized by basaltic cliffs originated by eruptions of Mt. Etna volcano (Fig. 9); in particular, the 4500 BC and 1669 AD lava flows extended to the north and south of the city, respectively, and poured into the sea^80^. The unique connection with the sea remained a small and narrow bay with a low sandy beach at the foot of the ancient city walls. This bay was initially used as natural harbor and successively filled by coastal fluvial sediments^59^. This peculiar configuration of the coastal topography enhanced the flooding, in particular the impact of the second wave that flooded the hinterland up to 510 m from the coastline (Fig. 9).

## Discussions—tsunami scenarios and related coastal flooding

The impact of the 1693 tsunami event determined significant damages and casualties along the coasts of south-eastern Sicily. Although several authors have speculated that the 1693 tsunami event was caused by a combined effect of fault displacement and submarine landslide^28,30^, the possibility that the 1693 event could have been the result of two distinct tsunami waves, one generated by the seafloor displacement and the other triggered by the consequent landslide, has never been considered and/or simulated. This scenario (DTS) is here modelled together with the ones of singular waves generated by fault (SFS) or landslide (SLS), and compared with geomorphological and historical constraints known in the area. Considering the short movement of the landslide along the MESC slope (Fig. 4) and its low deformation, a rigid model was applied to better simulate the tsunami wave generation due to landslide movement^81,82^. On the other hand, the rheological models applied in other contexts, such as in South China Sea^62^, considered larger volumes (from 10 km^3^ to 200 km^3^) and more extended areas (from 230 km^2^ to 1151 km^2^) than our study case. For these reasons, we decided to apply a rigid model for the generation of tsunami in response to the landslide movement, which provides greater tsunami waves than rheological models^83,84^.

The SFS scenario highlighted a significant wave height of about 6 m in the offshore, which increased after the breaker zone reaching a height of about 10 m (Fig. 6a). On the other hand, the tsunami modelled through SLS scenario showed a wave height of about 5 m in the offshore, which increased after the breaker zone reaching a height of about 6 m (Fig. 6b). However, the modelling of SFS and SLS scenarios provided underestimated flooding with respect to geological evidence, as observed in the Mascali and Augusta areas^54,55,85^. Both the SFS and SLS scenarios showed a withdrawal of about 45 m, but not the double withdrawal reported in historical documents^58,79,86^. The DTS scenario instead showed the propagation of two consecutive waves in offshore, the first with a height of about 12 m and the second with a height of about 9 m (Fig. 6c). Both waves in the DTS scenario showed an increase in wave height of about 13 m, with a double withdrawal in the proximity of the coasts.

The numerical modelling in Celeris environment showed a different extent of flooding following the aforementioned scenarios (Table 3), highlighting how the DTS determined greater extent of flooding than others. The sensitivity of the inundation for each scenario depends on the amplitude of the incoming wave and the topography of the coastal region. Gibbons et al.^71^ demonstrated that the sensitivity of the inundation area to the Manning value is likely to be greater when the topography increases slightly. However, for the low-lying areas, the sensitivity of inundation is less dependent to the Manning value^71,87^ where the coastal slope is the main driven factor of inland flooding. This appears to be in agreement with what was estimated by Scicchitano et al.^73^ along the coasts of south-eastern Sicily through the use of Terrestrial Laser Scanner technique.

According to Tonini et al.^30^ and Argnani et al.^28^, a tsunami due only to submarine slide may not have determined a flooding surface as reported in historical records. However, the geophysical surveys showed the presence of a submarine landslide of significant volume that, as also suggested by Paparo et al.^29^, can generate a high tsunami wave in deep water. Therefore, the dual mechanism of fault displacement and submarine landslide could represent a step-forward in resolving the source of tsunami of 1693 AD.

**Figure 10:**
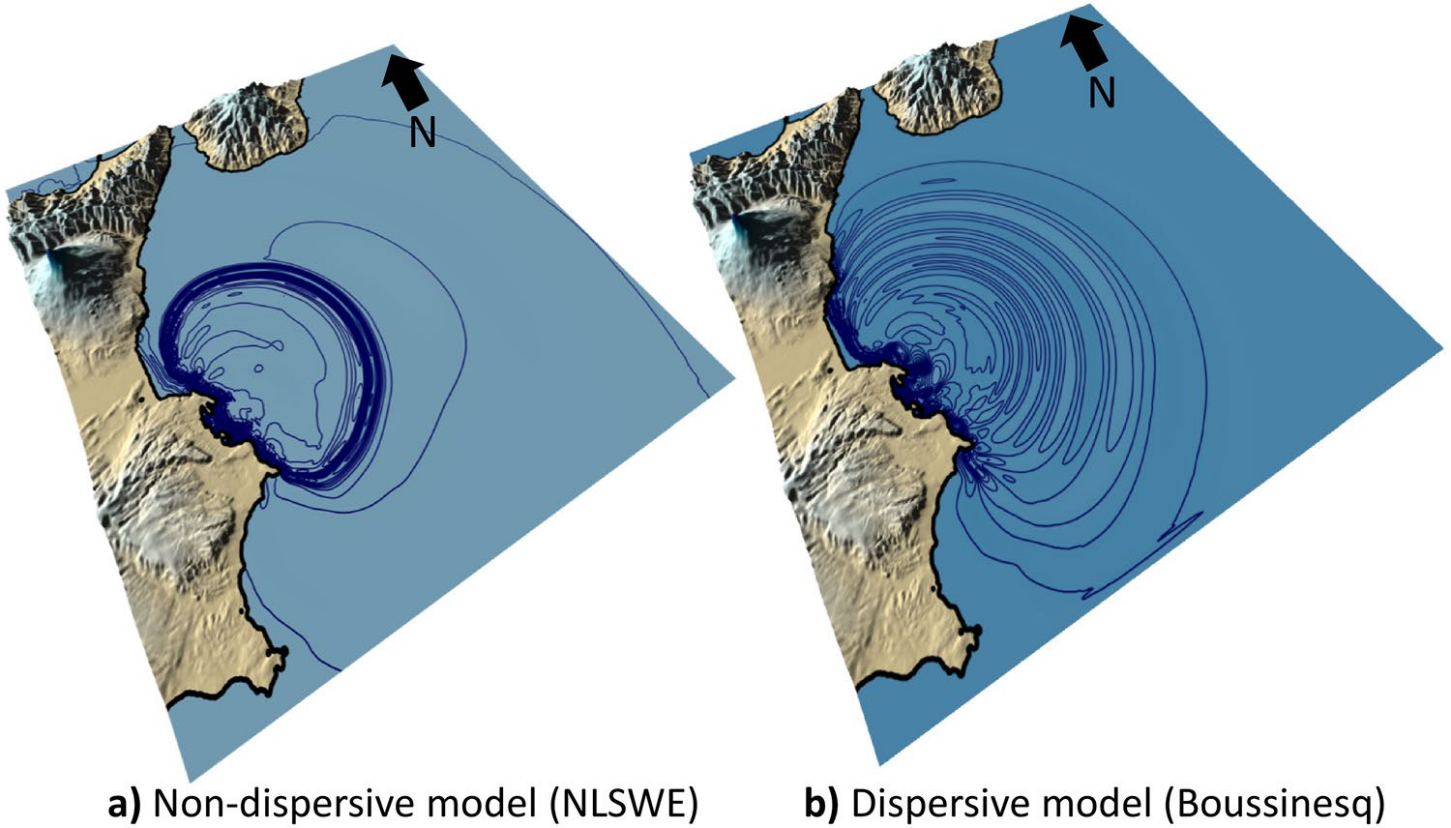
Dispersion effects for the tsunami propagation in a DTS scenario, 10 min after the start of simulation; a) non-dispersive model based on nonlinear shallow water equations (NLSWE); b) dispersive model based on Boussinesq equation. The maps were obtained by co-authors through QGIS—software (version 3.14.16); https://www.qgis.org/it/site/, license Creative Commons. Attribution-Share Alike 3.0 licence (CC BY-SA) integrated with ESRI World Imagery.

The Boussinesq model was compared with the non-dispersive model based on the nonlinear shallow water equations (NLSWE) applied for DTS scenario. The Boussinesq model includes the lowest order effects of frequency dispersion and nonlinearity, which can reproduce the soliton fission effects (consisting in the split of waves from tsunami crest) and the intensification of tsunami height. Furthermore, this behaviour was also observed in the airborne video footage taken at the northern Sendai Bay during the Tohoku earthquake tsunami^88^. On the other hand, NLSWE is not able to describe the frequency-dispersion behaviour for tsunami propagation (Fig. 10).

**Figure 11:**
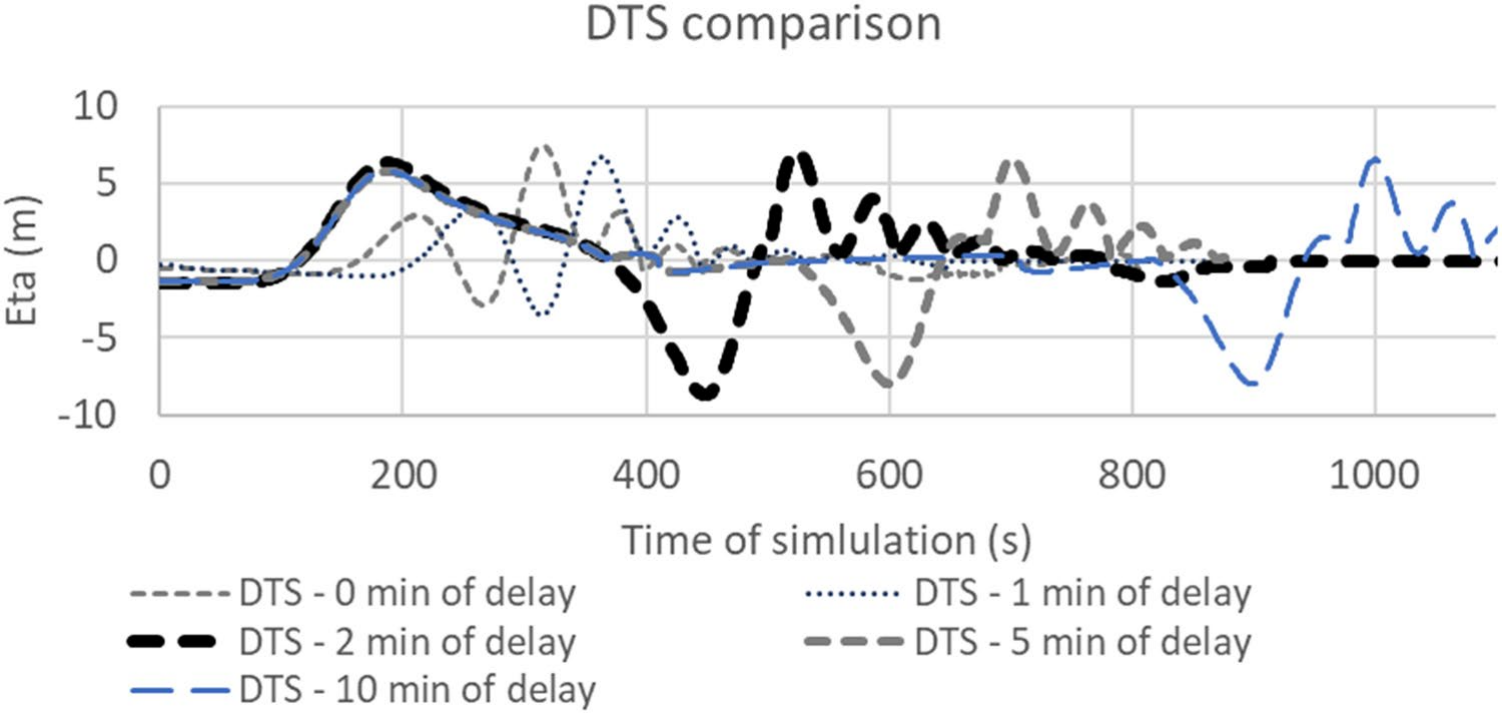
Comparison of sea-surface elevation (Eta) for DTS scenarios modelled on Catania area with different time delays between fault displacement and landslide movement.

Simulations with different time delays between fault displacement and landslide movements were performed to choose the more reliable scenario for the tsunami of 1693 AD. Modelling performed with 0 min and 1 min of delay showed a destructive interference of the waves, without the genesis of two consecutive waves impacting the coast (Fig. 11). Modelling performed with 2 min, 5 min, 10 min are more reliable for the simulation of the 1693 tsunami event, as they show two distinct waves divided by the negative sea-surface elevation due to mass deficit during the landslide movement. The delay of 2 min between earthquake and submarine landslide was chosen to reproduce the worst-case scenario, in which the combined wave fronts due to fault and landslide provide the greatest flooding surfaces. Since modelling with a simultaneous displacement of fault and landslide resulted in a destructive wave interference, due to the velocity of the landslide less than the seafloor deformation (Fig. 11), it is necessary to take into account the relatively slow motion of landslides and its delay with respect to the wave generated by fault displacement.

Another important evidence supporting the DTS scenario is the double withdrawal reported in historical documents along several coastal areas. The multi-withdrawals observed during tsunami events are usually connected to three main processes: (i) the different slip distribution during an earthquake^89,90^, (ii) the reflectivity of the coastal morphology impacted by tsunami wave fronts ^91^, (iii) the different triggers of waves during the tsunami event (different fault displacements, coastal landslides, submarine landslides, etc.)^30,66^. The spatial and temporal difference on the slip distribution was the trigger of tsunamis of Tohoku, 2011 AD^92,93^, where multi-withdrawals were observed in function of the different waves caused by distinct slips of sub-faults of Japan Trench. On the other hand, the coastal morphology can determine the tsunami reflection, as happened in Vathy Bay, NE Samos, 2020 AD^94^, and in the Sendai Plain, Japan, 2016 AD^91^. The multi-withdrawals are mainly attributed to multi-sources causing tsunamis, and many examples were reported for Sulawesi, Indonesia, 2018 AD^95^, where fault displacement and coastal landslides determined distinct tsunamis in the Palu Bay. In our study case, double withdrawal cannot be explained by the high reflectivity of the coastal morphology, because eastern Sicily is mainly represented by beach system with few promontories characterized by high cliff. Further, fault displacement analysis and slip tendency modelling performed on the MESC faults system^26^, revealed the F3 fault as a continuous structure (Figs. 4a, 5), prone to slip along its entire length. Accordingly, time-shifted along-fault multiple seafloor coseismic ruptures with double-wave generation are unexpected. As a consequence, only two distinct tsunami waves, generated by different sources, can properly explain the double withdrawal, up to 100 m, described by historical documents. On the other hand, this hypothesis is supported also by geological evidence detected in Ognina area (Scicchitano et al.^56^), where the deposits of the 1693 tsunami event are characterized by two distinct sub-units determined by a sequence of anomalous waves in a short temporal range. This is in agreement with the DTS scenario modelled with at least 2 min of delay between fault displacement and landslide movement.

### Comparison between results from tsunami modelling and historical chronicles

**Figure 12:**
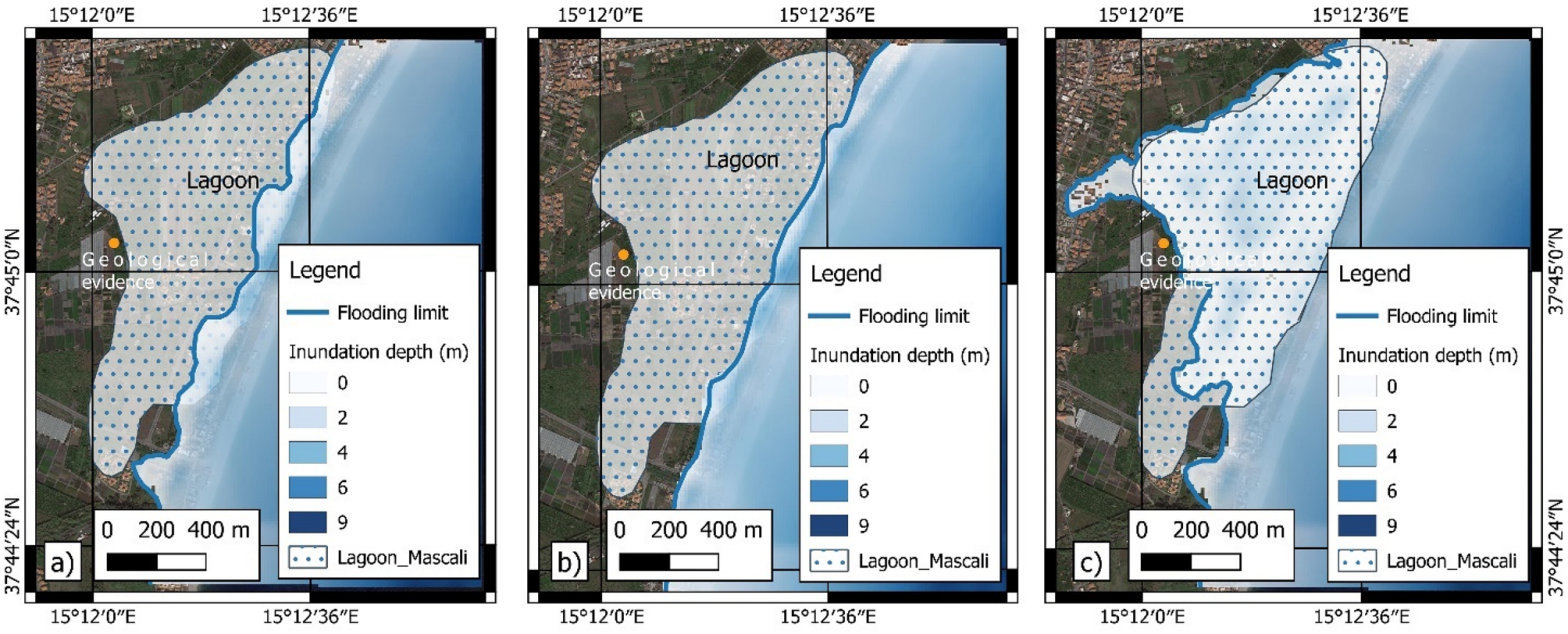
Modelling of tsunami inundation in the Mascali area; (**a**) SFS scenario; (**b**) SLS scenario; (**c**) DTS scenario. The maps were obtained by co-authors through QGIS—software (version 3.14.16); https://www.qgis.org/it/site/, license Creative Commons. Attribution-Share Alike 3.0 license (CC BY-SA) integrated with ESRI World Imagery.

Historical chronicles reported descriptions about the dynamics of the tsunami waves and related damages and casualties for the areas of Mascali, Catania, Augusta, Priolo-Thapsos, Siracusa and Ognina (Fig. 3). One of the most relevant effects reported in the chronicles of the 1693 tsunami event is the double withdrawal of the sea, described for the Mascali, Catania, Augusta and Siracusa areas. In the Mascali area, mostly in the proximity of Gurna lagoon (Fig. 12), a double withdrawal of about 800 m was reported by Boccone^58^ and Baratta^96^. In the Catania harbor, several historical documents reported a double withdrawal of about 100 m leaving boats stranded on the sea-floor^58,96,97^ and references therein. When the sea returned, the wave flung the boats beyond the walls, into the town. At Augusta and Siracusa^58^ the sea retreated of about 60 m and 100 m, respectively, then returned with a wave reaching heights of about 12 m^98^. Galley ships of the Knights of Malta, anchored in the harbor of Augusta, ran aground on the seabed due to the waves^96^. Our results highlighted that the double withdrawal described in historical sources for several sites is explainable only with the DTS scenario. Another important effect, that could be useful for the analyses of modelling results, is the maximum flooding reached by the 1693 AD tsunami. In addition to the information provided by historical reports, data can also be found in field as geomorphological and sedimentological records. The cores sampled in the Gurna lagoon, near Mascali showed the presence of four tsunami deposits at about 350 m from the shoreline dated at 365 AD, 1330 AD, 1169 AD, 1693 AD^54^ and references therein. Moreover, Boccone^58^ and Baratta^96^ reported a maximum flooding of 1500 m for this coastal area. The numerical models for the Mascali area suggest that the SFS and SLS scenarios are not able to inundate the entire surface of the ancient lagoon (Fig. 12a,b), while the DTS scenario shows a flooding extension of 1075 m (Fig. 12c), confirming the historical descriptions of Boccone^58^ and Baratta^96^.

**Figure 13:**
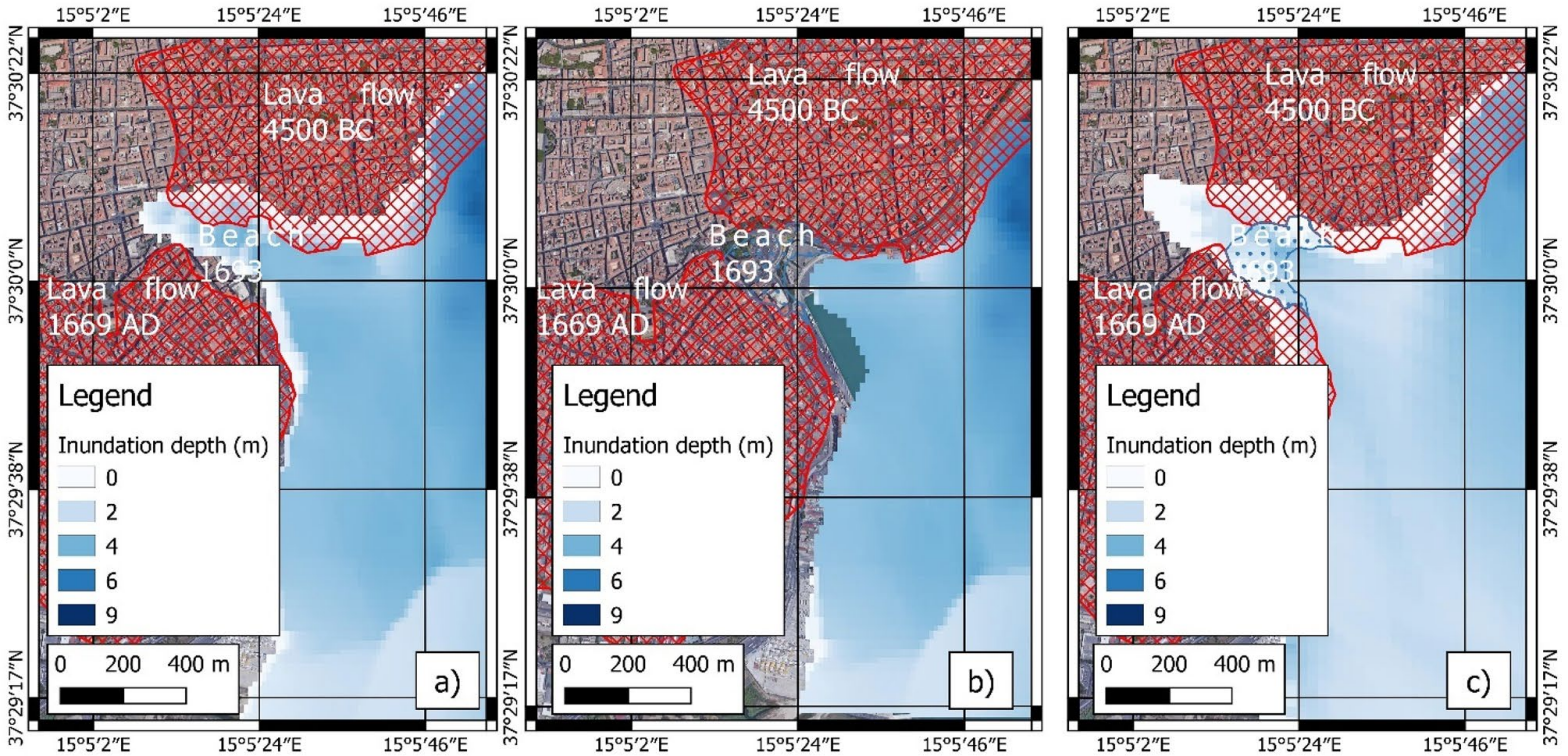
Modelling of tsunami inundation in the Catania area; (**a**) SFS scenario; (**b**) SLS scenario; (**c**) DTS scenario. The maps were obtained by co-authors through QGIS—software (version 3.14.16); https://www.qgis.org/it/site/, license Creative Commons. Attribution-Share Alike 3.0 licence (CC BY-SA) integrated with ESRI World Imagery.

Several studies in the past provided an accurate description of the 1693 tsunami impact on the Catania coast^25,58,96,99^, referring to the historical sources that described a double withdrawal up to 45 m from the coastline and a flooding extension up to 350 m landward. Probably, the extensive inland inundation was amplified by the different morphology of the Catania coastline with respect to the present-day^59^. The reconstruction in GIS environment of the paleo-landscape of Catania reported by historical sources^100,101^ highlighted the small sandy bay between two lava promontories as a preferential way for the wave propagation and inundation (Figs. 9, 13). In fact, the numerical model of Boussinesq highlighted the double withdrawal and the wave inundation enhanced by the small bay. This behavior is in agreement with the DTS scenario, in which the entity of the double withdrawal (on the order of about 100 m) could be explained with a first wave front generated by fault displacement and the consecutive wave generated by movement of the submarine landslide. The DTS scenario showed inland inundation larger than the SFS and SLS scenarios, with a flooding of about 350 m, as described by Boccone^58^ and Baratta^96^. Furthermore, as shown in Fig. 13b, the tsunami modelled with SLS scenario may not have been able to cause inland flooding from the small bay in front of the city of Catania.

**Figure 14:**
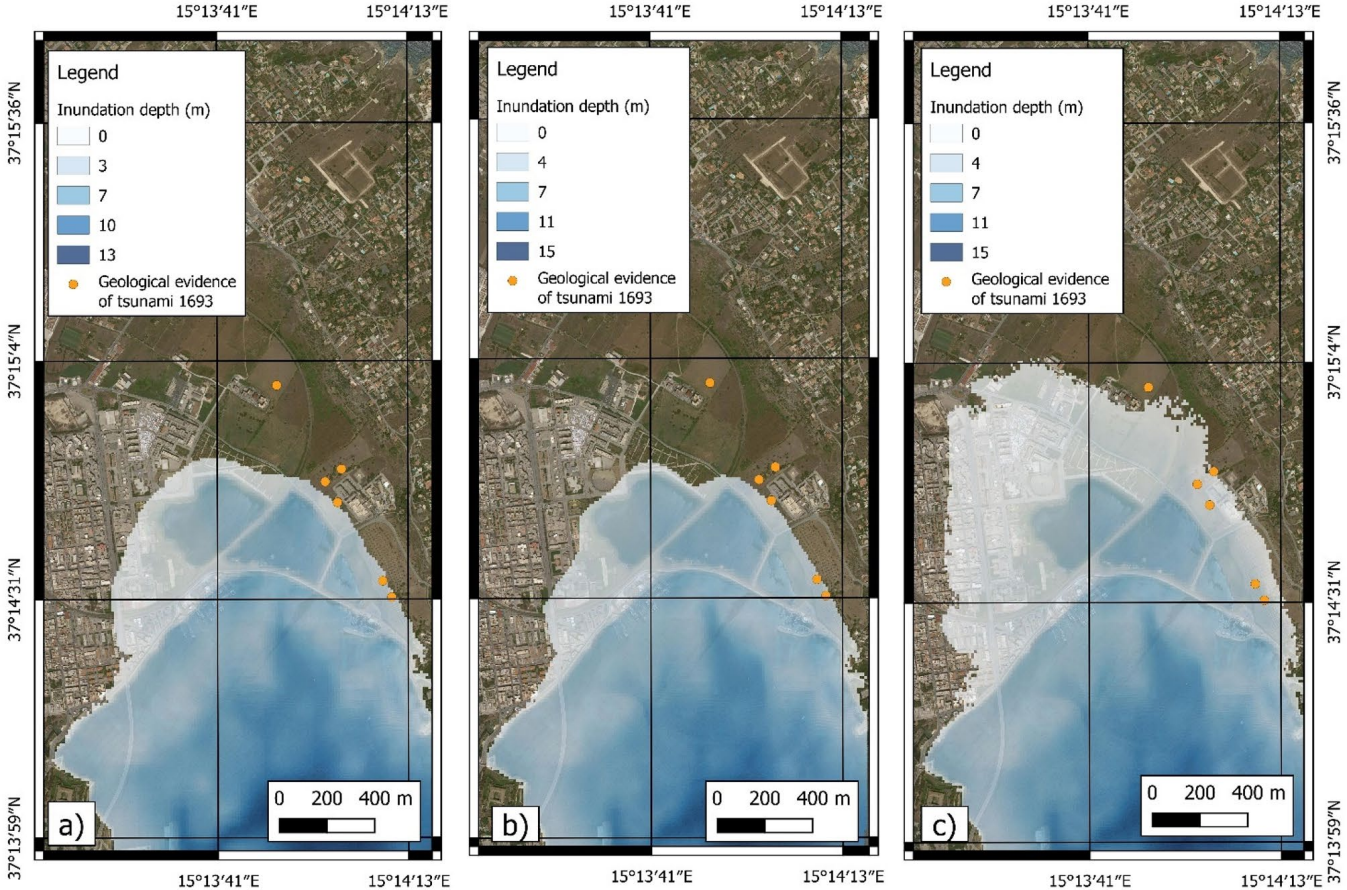
Modelling of tsunami inundation in the Augusta area; (**a**) SFS scenario; (**b**) SLS scenario; (**c**) DTS scenario. The maps were obtained by co-authors through QGIS—software (version 3.14.16); https://www.qgis.org/it/site/, license Creative Commons. Attribution-Share Alike 3.0 licence (CC BY-SA) integrated with ESRI World Imagery.

The Augusta area (Fig. 14) was subjected to the worst combined effect of earthquake and tsunami inundation during the 1693 AD event^58,96^ and references therein, with severe damages and local seismic amplification due to the unconsolidated sediments^25^. Here a double withdrawal of about 37–45 m was observed^58,96^, and a subsequent flooding involved the entire city of Augusta, reaching the church of Convento Dominicani, located 150 m far from the shoreline. The model results show that the best fit between the location of tsunami deposits^55^ and the inland flooding is only obtained with DTS scenario. In the southern part of the Augusta area (Fig. 14), in the Saline di Priolo Reserve, the best evidence was observed inside cores, sampled in the marsh zone, showing the presence of tsunami deposits attributed to the 1693 AD event^55^.

**Figure 15:**
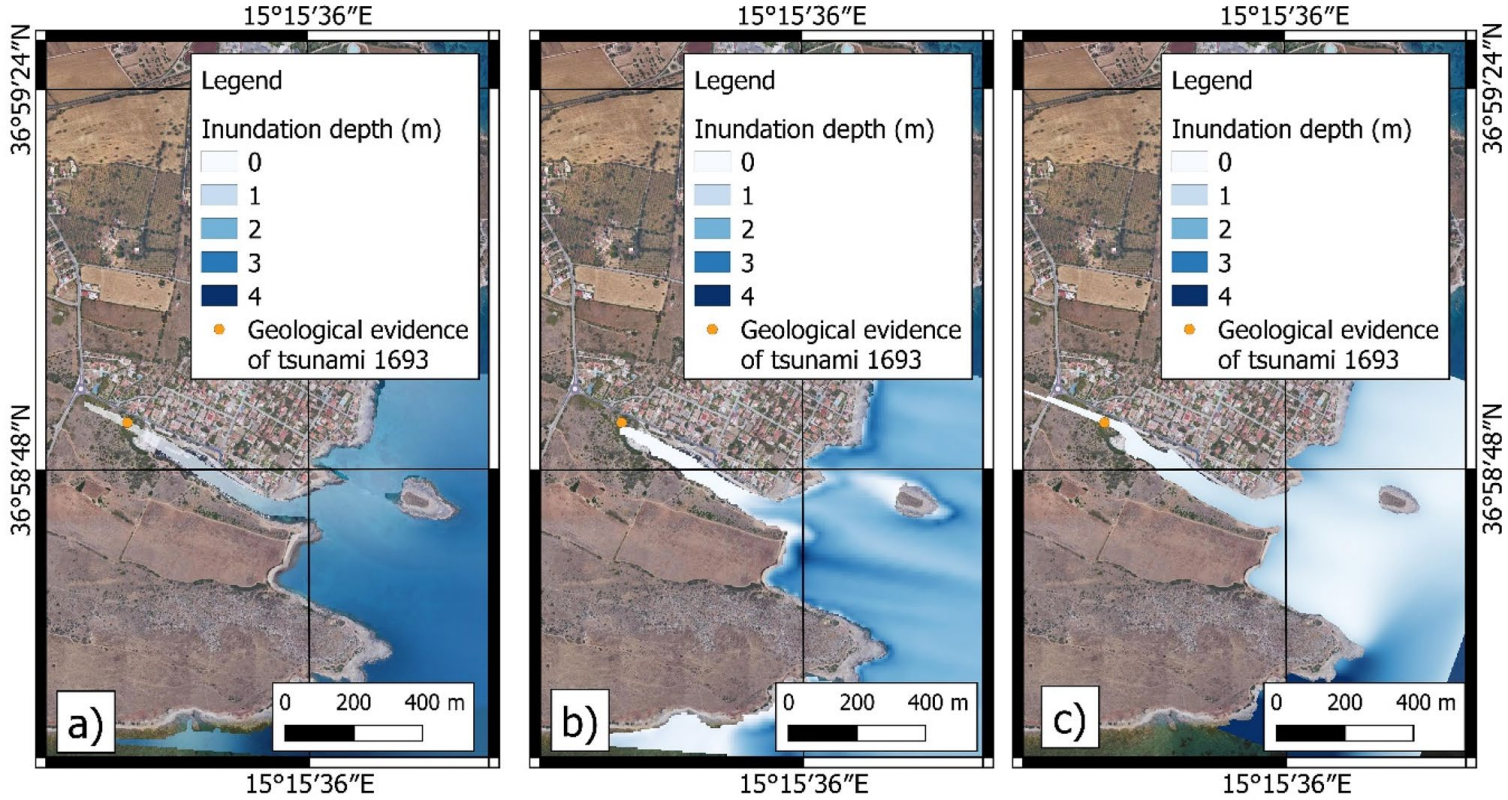
Modelling of tsunami inundation in the Ognina area; (**a**) SFS scenario; (**b**) SLS scenario; (**c**) DTS scenario. The maps were obtained by co-authors through QGIS—software (version 3.14.16); https://www.qgis.org/it/site/, license Creative Commons. Attribution-Share Alike 3.0 licence (CC BY-SA) integrated with ESRI World Imagery.

In the Ognina coastal area, only the SFS and DTS scenarios are able to explain the coarse deposits located inland at the end of the marine channel and attributed to tsunami events in previous studies^27,56^. In the case of the DTS scenario, the wave front able to inundate the inland surface was due to the first impulse, caused by the fault displacement, while the subsequent wave due to the submarine landslide was attenuated by the diffraction of the Maddalena Peninsula to the north-east (Figs. 3, 15).

## Conclusions—the most reliable tsunamogenic scenario

In this work, an in-depth numerical modelling of tsunami waves triggered by offshore fault displacement and submarine landslides was performed to obtain the more reliable scenario for the 1693 AD tsunami event in south-eastern Sicily. The bathymetric and seismic data clearly showed the presence of submarine landslides that could have been activated during the coseismic motion of the source fault. Considering this evidence, all possible scenarios of the 1693 tsunami event were modelled to simulate the tsunami propagation and the related inland flooding. The tsunami scenarios were simulated considering three tsunamigenic sources: the fault displacement (SFS), the submarine landslide movement (SLS), the dual mechanism of fault displacement and submarine landslide (DTS). Modelling has been focused on the main low coastal areas impacted by consecutive waves, which were Mascali, Catania, Augusta, Siracusa, Ognina. For each zone, the inland flooding was assessed considering the past topography reconstructed through historical documents. The joint analysis of modelling, historical documents and geological evidence highlighted the following points:

1. The SFS and SLS scenarios determined underestimated flooding in some areas, such as Mascali and Augusta, and did not match with geological evidence of 1693 AD tsunami event; the DTS scenario provided the best match with geological evidence and historical reports;
2. The DTS scenario is the only one that showed the double wave impact and the double withdrawal, according with descriptions reported in historical documents;
3. The simultaneous movement between fault displacement and slide determined two consecutive waves characterized by a destructive interference with related decrease in height, while a delay of 2 min between fault displacement and slide movement provided two consecutive impacting waves with maximum height.

Results imply the need to reevaluate the tsunami hazard and risk along the coasts of south-eastern Sicily, characterized by the presence of several towns (e.g. Catania, Augusta, Siracusa) and by one of the main petrochemical complexes in Italy.

## Materials and methods

Seismic profiles and bathymetric data available for the Western Ionian Basin^26,28,102^ were exploited to perform morpho-bathymetric analysis along the steep (950 m high) submarine slope of the MESC. The E-W oriented seismic profiles p605 and MESC 08 are from the RV Poseidon expedition POS496 dataset (R/V Poseidon, March–April 2016^103^) and from previous published paper^28^, respectively. They were acquired with different resolution, higher for the P605 and lower for the MESC08 line (Fig. 4). High-resolution bathymetric data come from a compilation of grid 2-arc second^102^.

Seismic data interpretation and 3D modelling have been performed within the Move 2019 geo-modelling software package (Petex Ltd). Seismic profiles (p605 and MESC08; Fig. 4a) were used to identify and map morpho-bathymetric features and their geometric parameters (e.g. landslide basal slip surface and paleo-bathymetric profile). The spatial extension of morpho-bathymetric features were instead constrained using the high-resolution bathymetry. Further, a grid of seafloor profiles (pseudo-sections) was extracted from the bathymetric data and used for a better mapping of the identified landslide deposit. To obtain a realistic 3D volume model and geometric parameters of the submarine mass deposit, seismic profiles were time/depth converted using the velocity model provided by Gambino et al.^26^ and references therein. Then, linear features picked along seismic profiles (i.e., the landslide basal slip surface) were interpolated via the ordinary Kriging algorithm to produce the 3D surfaces. Finally, the obtained surfaces were used to derive the volume of the considered features (Fig. 4f), from which the necessary parameters for tsunami simulation were extracted.

Tsunami modelling performed in this work consists of three steps: (i) the tsunami generation process in function of a specific tsunamogenic source, (ii) the propagation of the tsunami-generated waves in deep water, (iii) the inland inundation. The modelling regarded three distinct tsunami scenarios:

- Singular tsunami generated by fault displacement on the sea-floor (SFS);
- Singular tsunami generated by a submarine landslide triggered by the earthquake (SLS);
- Double tsunami generated by dual mechanism due to fault displacement and submarine landslide (DTS).

The triggering of the tsunami wave due to the displacement of the fault was modelled according to the Okada method^77^, where the initial wave conditions were assessed considering the fault parameters described by Gambino et al.^26^. Parameters of the Okada method are three angles orienting the “slip plane” (strike, dip, rake), fault length, width of the rupture area and Magnitude of the seismic event (Tables 1, 2).

The trigger of the tsunami wave due to the submarine landslide was reconstructed using the model of Grilli et al.^104^ and Grilli and Watts^105^, in which length, width and vertical displacement of the landslide must be inserted. Following this approach, the tsunami wave height is related to the landslide thickness and its wavelength is related to the landslide width. The landslide mass is simplified as a block and the model computes the motion of the center of the mass that moves along a predefined path subject to the action of the body forces (gravity and buoyancy), the bottom friction, the frontal drag and the block-seabed interactions. The landslide velocity reaches its maximum value in the first 60–90 s^105^.

The trigger of the tsunami wave due to the dual mechanism of fault displacement and submarine landslide was modelled by GEOWAVE that is a comprehensive tsunami simulation model obtained by combining the Tsunami Open and Progressive Initial Conditions System (TOPICS) with the fully non-linear Boussinesq wave model (FUNWAVE)^60,106^. Different delay between fault displacement and submarine landslide movement have been tested to model the double impact of waves. A delay of 2 min was inserted between the trigger of the landslide and the fault displacement for the DTS scenario. This delay was chosen to avoid the destructive interference that can be generated by the simultaneous motion of the wave due to fault displacement and the wave due to slide movement.

The tsunami propagation, from deep to shallow waters, was performed through GEOWAVE (Table 4). The wave dynamics at different spatial scales was obtained by the use of different grid resolutions. For this reason, the tsunami propagation in deep water was performed using a coarse grid with 120 × 120 m cells, while the tsunami propagation in shallow water was performed using a finer grid with 4 × 4 m cells (Fig. 5b,c). For both grids, bathymetric data from MBES, nautical maps and topographic data from LiDAR surveys were interpolated.

**Table 4:** Parameters of tsunamogenic sources used for GEOWAVE modelling.

Tsunamogenic sources	Mean strike	Mean dip (°)	Length (km)	Co-seismic seafloor rupture (m)	Width (m)	Mw
Fault (Gambino et al.^26^)	N352E	49	56.46	2.3	5.275	7.4

Submarine landslide—deposit 1	Longitude—UTM 33N	Latitude—UTM 33N	Length (m)	Thickness (m)	Density (kg/m^3^)	Width (m)
	537,534	4,126,287	4300 ± 25	65 ± 5	2400 ± 25	2700 ± 25

In order to compare the tsunami effect on the coastal area with chronicles reported in historical documents^58,59,79,96,97,107^ and references therein, a modelling of the coastal inundation was performed. For each scenario, the coastal flooding was assessed in Celeris environment^69,70^ using the wave amplitude and the wave period modelled in shallow water.

GEOWAVE simulates tsunami generation and propagation using a 4th order fully nonlinear and fully dispersive Boussinesq wave model with multiple wave dissipation mechanisms, wave breaking, and dry land overflow. The model of GEOWAVE is based on the fully nonlinear Boussinesq equation^108^, expressed as follow:

$$\eta + \nabla \left\{ {\left( {h + \eta } \right)\left[ {u_{\alpha } + \left( {z_{\alpha } + 0.5\left( {h - \eta } \right)} \right)\nabla \left( {\nabla \cdot \left( {hu_{\alpha } } \right)} \right) + \left( {0.5z_{\alpha }^{2} - \frac{1}{6}\left( {h^{2} - h\eta + \eta^{2} } \right)} \right)\nabla \left( {\nabla \cdot u_{\alpha } } \right)} \right]} \right\} = 0$$

$$\begin{aligned} & u_{\alpha t} + \left( {u_{\alpha } \cdot \nabla } \right)u_{\alpha } + g\nabla \eta + z_{\alpha } \left\{ {0.5z_{\alpha } \nabla \left( {\nabla \cdot u_{\alpha t} } \right) + \nabla \left( {\nabla \cdot \left( {hu_{\alpha t} } \right)} \right)} \right\} \\ & \quad + \nabla \left\{ {0.5\left( {z_{\alpha }^{2} - \eta^{2} } \right)\left( {u_{\alpha } \cdot \nabla } \right)\left( {\nabla \cdot u_{\alpha } } \right) + 0.5\left[ {\nabla \cdot \left( {hu_{\alpha } } \right) + \eta \nabla \cdot u_{\alpha } } \right]^{2} } \right\} \\ & \quad + \nabla \left\{ {\left( {z_{\alpha } - \eta } \right)\left( {u_{\alpha } \cdot \nabla } \right)\left( {\nabla \cdot \left( {hu_{\alpha } } \right)} \right) - \eta \left[ {0.5\eta \nabla \cdot u_{\alpha t} + \nabla \cdot \left( {hu_{\alpha t} } \right)} \right]} \right\} = 0 \\ \end{aligned}$$

where η is the sea-surface elevation, h is the still water depth, u_a_ is the horizontal velocity vector at the water depth z = z_a_ =  − 0.531 h, $\nabla$ is the horizontal gradient operator, g is the gravitational acceleration, and subscript *t* is the partial derivative with respect to time.

On the other hand, the Celeris model is based on the governing Boussinesq equations modified by Madsen and Sørensen^109^:

$$U_{t} + F\left( U \right)_{x} + G\left( U \right)_{y} + S\left( U \right) = 0$$

$$U = \left[ {\begin{array}{*{20}c} h \\ P \\ Q \\ \end{array} } \right],\quad F\left( U \right) = \left[ {\begin{array}{*{20}c} P \\ {\frac{{P^{2} }}{h} + \frac{{gh^{2} }}{2}} \\ \frac{PQ}{h} \\ \end{array} } \right],\quad G\left( U \right) = \left[ {\begin{array}{*{20}c} Q \\ \frac{PQ}{h} \\ {\frac{{Q^{2} }}{h} + \frac{{gh^{2} }}{2}} \\ \end{array} } \right],\quad S\left( U \right) = \left[ {\begin{array}{*{20}c} 0 \\ {ghz_{x} + \varphi_{1} + f_{1} } \\ {ghz_{y} + \varphi_{2} + f_{2} } \\ \end{array} } \right]$$

where U is the conservative variables vector, F(U) and G(U) are the advective flux vectors, and S(U) is the source term that includes bottom slope, friction, and dispersive terms; h is the total water depth; P and Q are the depth-integrated mass fluxes in x and y directions respectively, where the x–y plane makes the horizontal solution field. Subscripts x and y denote spatial differentiation, with respect to the corresponding direction, and subscript *t* denotes temporal differentiation. z is the bottom elevation measured from a fixed datum. f_1_ and f_2_ are the bottom friction terms and g is the gravitational acceleration coefficient. ${\varphi }_{1}$ and ${\varphi }_{2}$ are the modified dispersive terms defined by Tavakkol and Lynett^69^.

**Figure 16:**
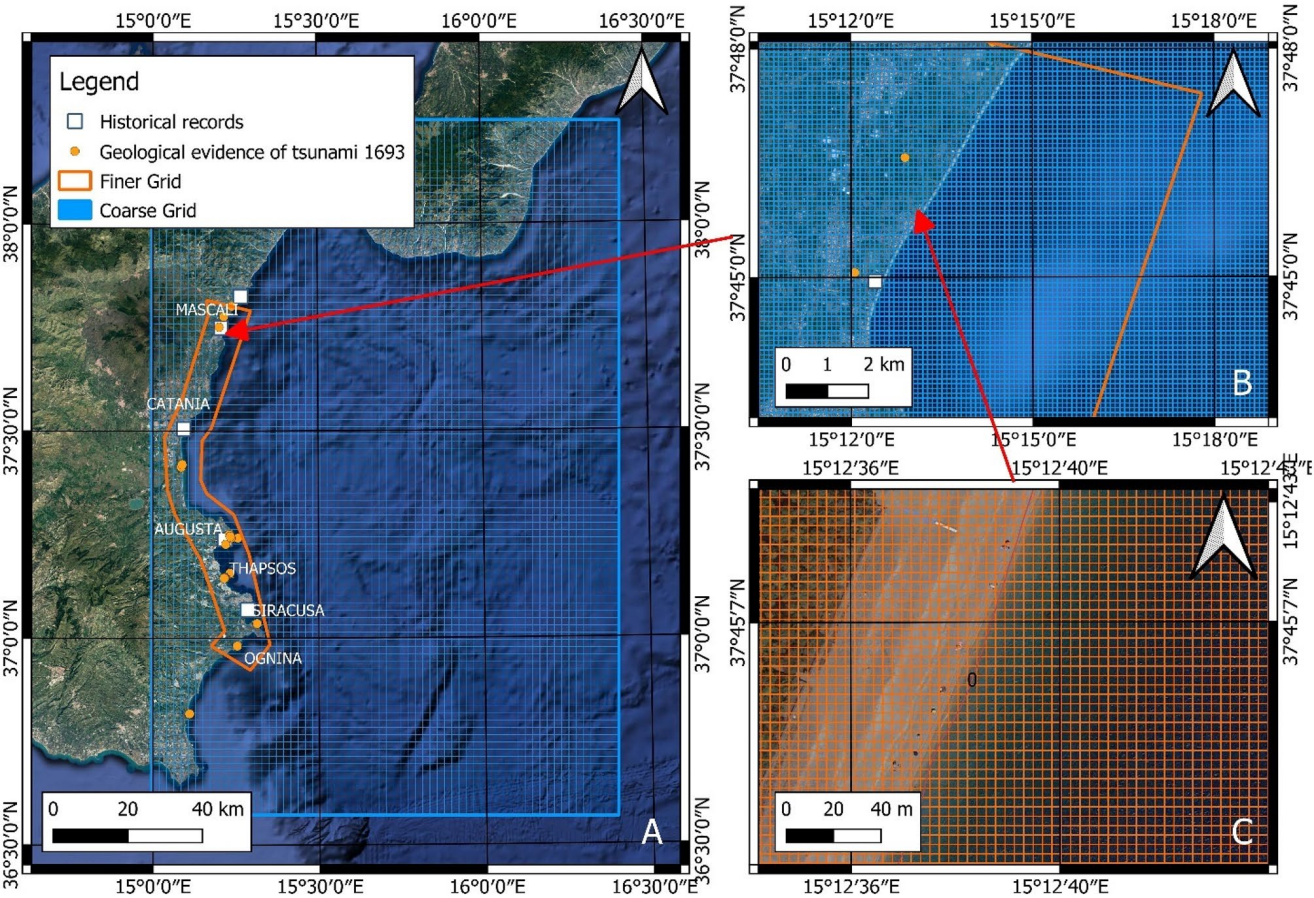
Resolution of the grids used for the simulation of tsunami events: (**A**)—extension of the coarse (blue rectangle) and finer (orange area) grids in eastern Sicily; (**B**)—cell resolution of the coarse grid in the Mascali area; (**C**)—cell resolution of the finer grid in the Mascali area. The maps were obtained by co-authors through QGIS—software (version 3.14.16); https://www.qgis.org/it/site/, license Creative Commons. Attribution-Share Alike 3.0 licence (CC BY-SA) integrated with ESRI World Imagery.

The coastal flooding was assessed in the following areas: Mascali, Catania, Augusta, Priolo-Thapsos, Siracusa, Ognina (Fig. 16).

### Reconstruction of the past topography

LiDAR data of *Ministero dell’Ambiente*^110^ were used to reconstruct the topography of the areas of Mascali, Catania, Augusta, Priolo-Thapsos, Siracusa and Ognina (see Supplementary Information). Based on the geological and geomorphological evidence described on historical maps, we obtained Digital Terrain Models (DTMs), with a grid cell width of 2 × 2 m, reproducing the possible coastal landforms of some places in the period of the 1693 AD tsunami.

At the end of the 17th century, with a mean sea level of about 0.27 m^27^, the Mascali coastal area was characterized by a well-defined mobile coastal system (“Plaia di Mascari”) and a back-dune lagoonal area extended for about 1 km landward, named Gurna^111^. The Catania area showed a different coastal landscape in 1693 AD: the town was surrounded to the West by a basaltic lava field, erupted few years before the 1693 tsunami event; the emplacement of the lava flow gave rise to a large promontory isolating a small sandy beach at the foot of the old city walls, and known as “Spiaggia della Marina”^59^.

The Augusta and Priolo-Thapsos areas were characterized by lagoonal/marsh environments^55^. These sites have been surveyed and filtered from the recent longshore interventions, breakwater and the current harbor, through LiDAR techniques. Furthermore, the current industrial facilities facing on the coasts have been deleted from the LiDAR data in order to reconstruct the past landscape, submerged during the 1693 tsunami event.

The reconstruction of the Ognina topography at the moment of the 1693 tsunami impact was obtained by the description of Spannocchi^112^, integrated with the surveys of Scardino et al.^27^. The river channel characterizing the Ognina area, with mouth located in correspondence of the current harbor, enhanced the inland inundation during the 1693 tsunami impact^27,56^.

## Supplementary Information

Supplementary Information.

## Data Availability

The datasets used and/or analysed during the current study available from the corresponding author on reasonable request.
