# Supplementary material for: The enigmatic 1693 AD tsunami in the eastern Mediterranean Sea: new insights on the triggering mechanisms and propagation dynamics
Source: Sci Rep. 2022 Jun 10;12:9573. doi: 10.1038/s41598-022-13538-x (PMC9187684; doi:10.1038/s41598-022-13538-x)
Supplement: Supplementary file 1 — Supplementary Information. [file 41598_2022_13538_MOESM1_ESM.docx]

**Past topography reconstructions**

LiDAR data of Ministero dell’Ambiente (“Progetto PST - Dati Lidar,” 2021) were processed to reconstruct the topography of the areas of Mascali, Catania, Augusta, Priolo, Siracusa and Ognina (Fig. S1) in the period of the tsunami of 1693. Based on the geological and geomorphological evidence described on historical maps, we obtained Digital Terrain Models (DTMs), with a grid cell width of 2x2 m, that have been processed in GIS environment to reconstruct the past landforms and landscapes (Fig.S2).


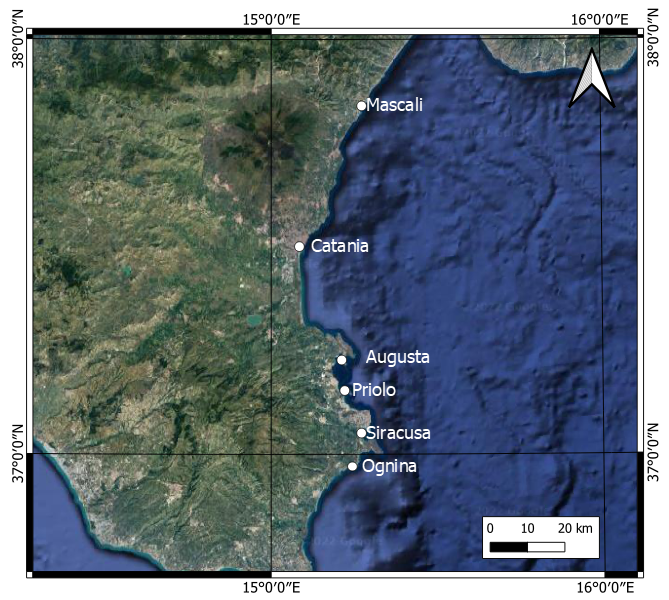


Fig. S1 – Sites of Sicily where palaeo-topographies have been reconstructed through geological evidence and historical records. The map was obtained by co-authors through QGIS—software (version 3.14.16); https://www.qgis.org/it/site/, license Creative Commons. Attribution-Share Alike 3.0 licence (CC BY-SA) integrated with ESRI World Imagery.


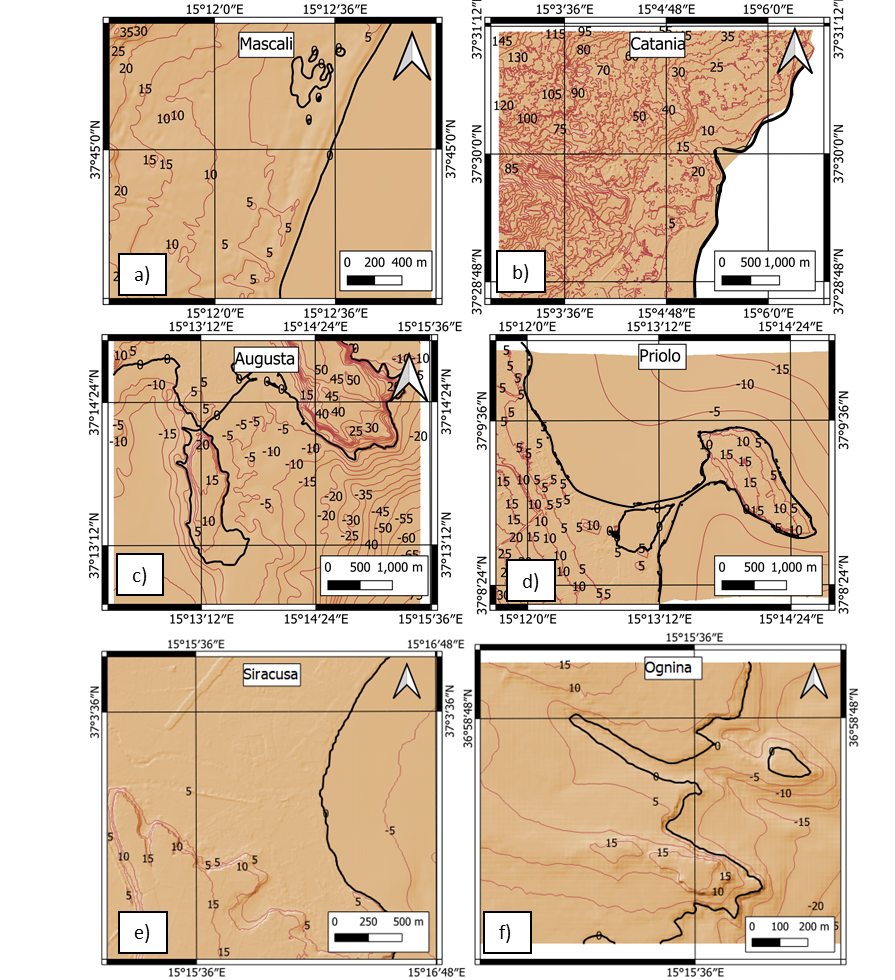


Fig. S2 – Palaeo-topographies reconstructed for the following areas: a) Mascali; b) Catania; c) Augusta; d) Priolo; e) Siracusa; d) Ognina. The maps were obtained by co-authors through QGIS—software (version 3.14.16); https://www.qgis.org/it/site/, license Creative Commons. Attribution-Share Alike 3.0 licence (CC BY-SA) integrated with ESRI World Imagery.
